# Impact of the changes in substrate specificity of herpes simplex virus 1 protein kinase Us3 on viral infection *in vitro* and *in vivo*

**DOI:** 10.1128/jvi.00400-25

**Published:** 2025-06-30

**Authors:** Saori Shio, Akihisa Kato, Jurika Kawasaki, Kousuke Takeshima, Yuhei Maruzuru, Naoto Koyanagi, Hayato Harima, Yasushi Kawaguchi

**Affiliations:** 1 Division of Molecular Virology, Department of Microbiology and Immunology, The Institute of Medical Science, The University of Tokyo, Minato-ku, Tokyo, Japan; 2 Department of Infectious Disease Control, International Research Center for Infectious Diseases, The Institute of Medical Science, The University of Tokyo, Minato-ku, Tokyo, Japan; 3 Research Center for Asian Infectious Diseases, The Institute of Medical Science, The University of Tokyo, Minato-ku, Tokyo, Japan; 4 PRESTO, Japan Science and Technology Agency (JST), Kawaguchi, Japan; 5 Laboratory of Veterinary Public Health, Faculty of Agriculture, Tokyo University of Agriculture and Technology, Fuchu, Tokyo, Japan; 6 The University of Tokyo, Pandemic Preparedness, Infection and Advanced Research Center, Tokyo, Japan; University of Toronto, Toronto, Ontario, Canada

**Keywords:** HSV-1, Us3, phosphorylation integrity, pathogenicity, protein kinase, activation loop

## Abstract

A serine-threonine protein kinase (PK), Us3, encoded by herpes simplex virus 1 (HSV-1), shares substrate specificity with host cellular PKs, protein kinase A (PKA), and AKT. Many Us3 substrates have been identified, and it is thought that during HSV-1 infection, Us3 fine-tunes the phosphorylation levels of individual substrates within their repertoire. However, the significance of this regulatory fine-tuning by Us3 during HSV-1 infection is poorly understood. Here, we found alanine at position 326 (Ala-326) in Us3 was required for the proper fine-tuning of Us3-mediated phosphorylation across the target repertoire in HSV-1-infected cells. Using recombinant viruses in which Us3 Ala-326 was replaced with valine (A326V) or isoleucine (A326I), we showed these mutations selectively altered the phosphorylation of only a subset of at least 14 Us3 target proteins tested in HSV-1-infected cells, with each mutation generally affecting different targets. Of note, (i) both mutations significantly reduced plaque sizes without affecting viral replication in cell cultures; (ii) the Us3 A326I mutation impaired viral replication in the brains of mice and improved survival following intracranial infection, whereas the Us3 A326V mutation had little effect; and (iii) the Us3 A326V mutation reduced ocular pathogenic manifestations and viral replication in the trigeminal ganglia and brains of mice, thereby improving survival following ocular infection. Taken together, these results suggest that the proper fine-tuning of Us3-mediated phosphorylation across its target repertoire is required for efficient cell-to-cell spread of HSV-1 *in vitro*, and its replication and pathogenicity *in vivo*.

**IMPORTANCE:**

The activation loop (A-loop) is a conformationally flexible loop that critically regulates cellular protein kinases (PKs), but its role in viral PKs during infection remains unclear. We demonstrated alanine at position 326 (Ala-326) in the A-loop of herpes simplex virus 1 (HSV-1) PK Us3 was important for the proper fine-tuning of Us3-mediated phosphorylation across the target repertoire in HSV-1-infected cells. This fine-tuning was necessary for efficient HSV-1 cell-cell spread in cell cultures, and replication and pathogenicity in mice. Taken together, fine-tuning phosphorylation levels of individual Us3 targets within its repertoire is important for HSV-1 infection *in vitro* and *in vivo*. Different amino acid substitutions at Us3 Ala-326 selectively affected the phosphorylation of most distinct Us3 targets, leading to varied phenotypic outcomes in viral replication and pathogenicity in mice. These results provide important clues to elucidate the mechanisms by which Us3 regulates HSV-1 infection *in vivo*.

## INTRODUCTION

Protein kinases (PKs) catalyze the transfer of phosphate groups from ATP to target proteins, a process known as phosphorylation (1). This is important for regulating numerous cellular processes, achieved by influencing protein stability, subcellular localization, protein activity, and intracellular trafficking (2, 3). The activation loop (A-loop), a dynamic structural element within the catalytic domain of PKs, is located between the DFG (Asp-Phe-Gly) motif at the N-terminal end and the APE (Ala/Pro/Ser-Pro-Glu) motif at the C-terminal end in many PKs (4, 5). It has a critical role in the regulation of kinase activity and substrate specificity, and it often contains one or more conserved phosphorylation sites, which are essential for the transition from an inactive to active conformation (6, 7). Specific residues within the A-loop interact directly with amino acids near the substrate’s phosphorylation site, forming hydrogen bonds, hydrophobic contacts, and/or electrostatic interactions (8, 9). These interactions enable PKs to selectively recognize substrates with specific amino acid sequences or structural features (1, 10).

The Us3 serine-threonine PK, encoded by herpes simplex virus 1 (HSV-1), is conserved among members of the subfamily *Alphaherpesvirinae* of the family *Herpesviridae* (11, 12). The phosphorylation motif of Us3 was identified as RnX(pS/T)YY, where “n” is greater than or equal to 2, “X” represents any amino acid, “pS/T” refers to phosphorylated serine or threonine, and “Y” denotes any amino acid except for acidic residues (13–15). This motif closely resembles those of cellular PKs including protein kinase A (PKA) and protein kinase B (PKB, also known as AKT) (16–19). The PK activity of Us3 was shown to be important for HSV-1 replication and pathogenicity based on studies that recombinant viruses encoding catalytically inactive Us3, as well as recombinant Us3-null mutant viruses, exhibit impaired growth properties in cell cultures and reduced virulence, pathogenic manifestations, and replication in mice (20–26). The activity of Us3 has multiple roles in HSV-1 infection, including the regulation of apoptosis and autophagy (16, 27, 28), intracellular trafficking of viral proteins and capsids (20, 23, 29–34), expression and translation of mRNA (18, 35), the morphology of infected cells (19, 36, 37), the activities of viral enzymes (37, 38), and host immune responses (39–44). Furthermore, previous studies have identified numerous Us3 substrates, including the viral proteins glycoprotein B (gB), UL31, UL34, VP13/14, viral nucleic acid metabolism enzyme dUTPase (vdUTPase), ICP22, Us3, Us9 (45), as well as host proteins including Beclin1 (16), Lamin A/C (46), IRF3 (42), BAD (47), kinesin family member 3A (KIF3A) (43), and tuberous sclerosis complex 2 (TSC2) (18). Some of these substrates, including Us3 phosphorylation at threonine 887 (Thr-887) in the major envelope gB (48), serine 77 (Ser-77) in the major tegument protein VP13/14 (31), Ser-147 in Us3 itself (24, 37), and Ser-187 in vdUTPase (38), have been linked to HSV-1 replication and pathogenicity *in vivo*.

**Fig 1:**
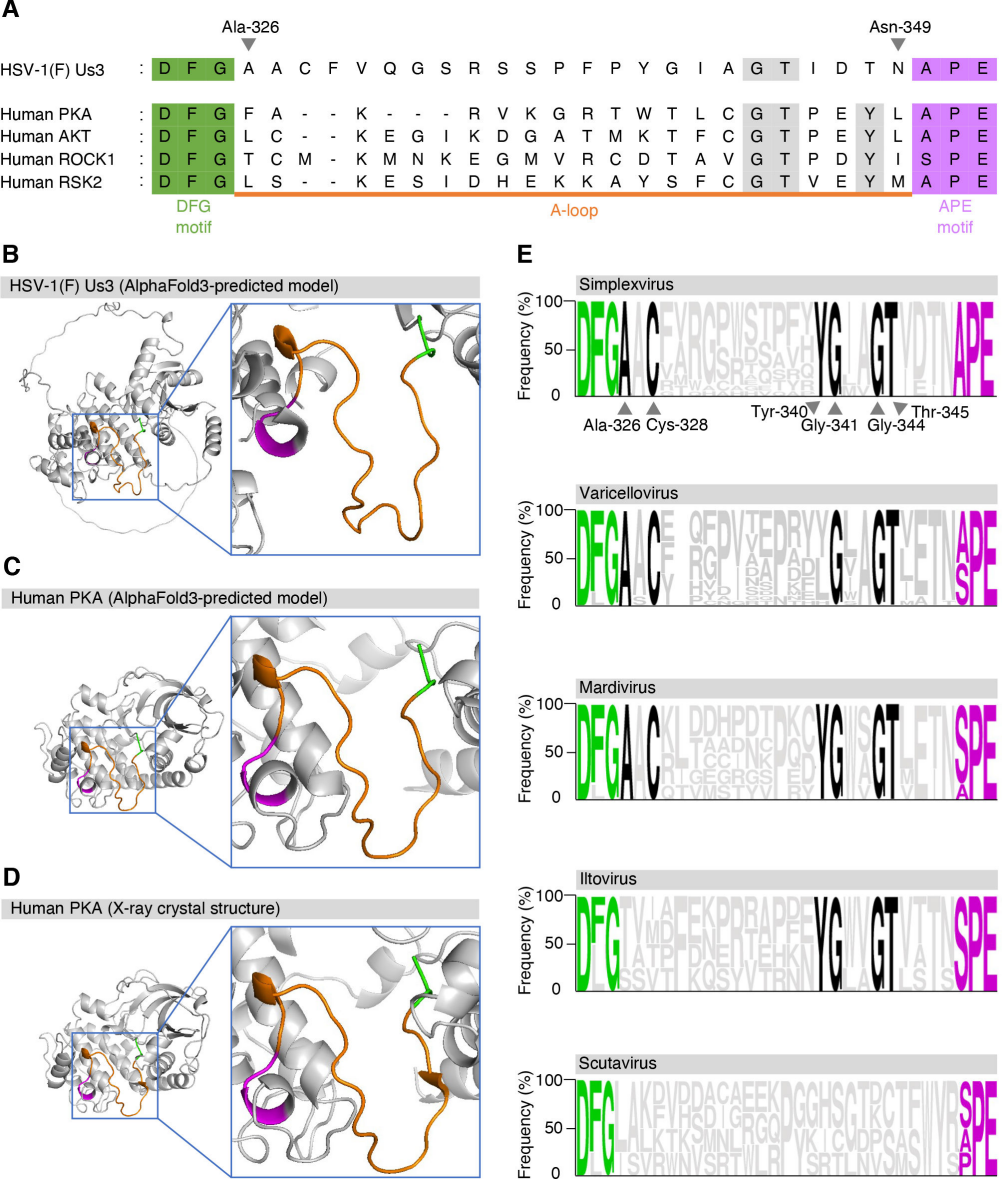
Comparative analysis of conserved motifs and A-loop regions in HSV-1 Us3 and related kinases. (**A**) Alignment of the amino acid sequences of HSV-1(F) Us3, and human PKA, AKT, ROCK1, and RSK2. Residues corresponding to the DFG motif are shaded in green, and those of the APE motif (SPE or PPE) are shaded in purple. The region corresponding to the A-loop is underlined in orange. (**B and C**) Predicted structures of full-length HSV-1(F) Us3 (**B**) and human PKA (**C**), modeled by AlphaFold3, including zoomed-in views highlighting their A-loop regions. (**D**) X-ray crystal structure of human PKA (PDB ID: 1ATP), with zoomed-in views highlighting its A-loop region. MnATP, a non-hydrolyzable ATP analog modeled in the ATP-binding site to stabilize the ATP-bound conformation of PKA, and a peptide inhibitor have been removed to improve clarity. (**E**) WebLogo representation of Us3 homologs from different genera of alpha-herpesviruses. The DFG and APE motifs are indicated in green and purple, respectively. Conserved amino acids shared by three or more genera are highlighted in black.

Accumulating evidence indicates that cellular PKs fine-tune the phosphorylation levels of individual substrates within their repertoire. Dysregulation of the fine-tuning of overall phosphorylation within the substrate repertoire of PKs, such as mitogen-activated protein kinases (MAPKs) and AKT, has been linked to cancer progression (49–51). Furthermore, various mutations in the A-loop of the lymphocyte-specific protein tyrosine kinase (LCK) selectively alter the phosphorylation levels of a specific subset of its substrates while excluding others, thereby influencing cellular transformation (52). These observations highlight that the fine-tuning of phosphorylation across the substrate repertoire of PKs is critical for the physiological functions of PKs. In contrast, although viral PKs such as HSV-1 Us3 were reported to regulate various host cellular and viral machinery, potentially by phosphorylating various substrates, the impact of the fine-tuning of their phosphorylation across the substrate repertoire on viral infection remains to be elucidated. This lack of knowledge has arisen because it is not known how viral PKs orchestrate phosphorylation across their substrate repertoire. To address this issue, the current study attempted to identify a residue in HSV-1 Us3 required for the fine-tuning of phosphorylation of this viral PK. Specifically, we focused on residues within the A-loop of HSV-1 Us3, paying particular attention to the residue immediately downstream of the DFG motif (DFG + 1 residue) because residues at the DFG + 1 positions in yeast and human serine-threonine PKs are associated with phosphoacceptor preference for serine or threonine (1, 53). Bulky residues are more common in this position in serine-selective kinases, whereas β-branched residues are more typical in threonine-selective kinases (1, 53). In contrast, phosphoacceptor preference can vary for some DFG + 1 residues such as Leu and Ala, which might be dependent on the structural and functional properties of the PKs (1, 53). The DFG + 1 residue in HSV-1 Us3 is Ala at position 326 (Ala-326), which is conserved among all examined members of the genera *Simplexvirus, Mardivirus*, and *Varicellovirus* within the subfamily *Alphaherpesvirinae* (Fig. 1). Given its high conservation and positioning at the DFG + 1 site within the Us3 A-loop, we hypothesized that Us3 Ala-326 is required for the fine-tuning of Us3-mediated phosphorylation across its substrate repertoire. In this study, we generated recombinant viruses encoding mutant Us3, in which Ala-326 at the DFG + 1 position was replaced with residues that differ from alanine in hydrophobicity and side-chain size to test this hypothesis and investigate a potential link between the fine-tuning of Us3-mediated phosphorylation across its substrate repertoire and HSV-1 infection in cell cultures and mice.

## RESULTS

### Predicted structural features of HSV-1 Us3

Amino acid alignment of HSV-1 Us3 and cellular PKs showed that Us3 contains conserved DFG and APE motifs in its amino acid sequence (Fig. 1A), suggesting that the A-loop of Us3 constitutes the region spanning Ala-326 to asparagine at position 349 (Asn-349) (Fig. 1A). AlphaFold3 structural modeling (54) predicted that this region would form loop structures (Fig. 1B), similar to the A-loop of PKA (Fig. 1C and D). Ala-326 at the DFG + 1 position in Us3 is fully conserved in members of the genera *Simplexvirus*, *Mardivirus*, and *Varicellovirus* registered by the International Committee on Taxonomy of Viruses (ICTV) (Fig. 1E), suggesting the importance of this amino acid residue. We hypothesized that Ala-326 in Us3 was required for the proper fine-tuning of Us3-mediated phosphorylation across its substrate repertoire as described above.

### Construction of recombinant viruses carrying amino acid substitutions at Ala-326 within the A-loop of Us3

**Fig 2:**
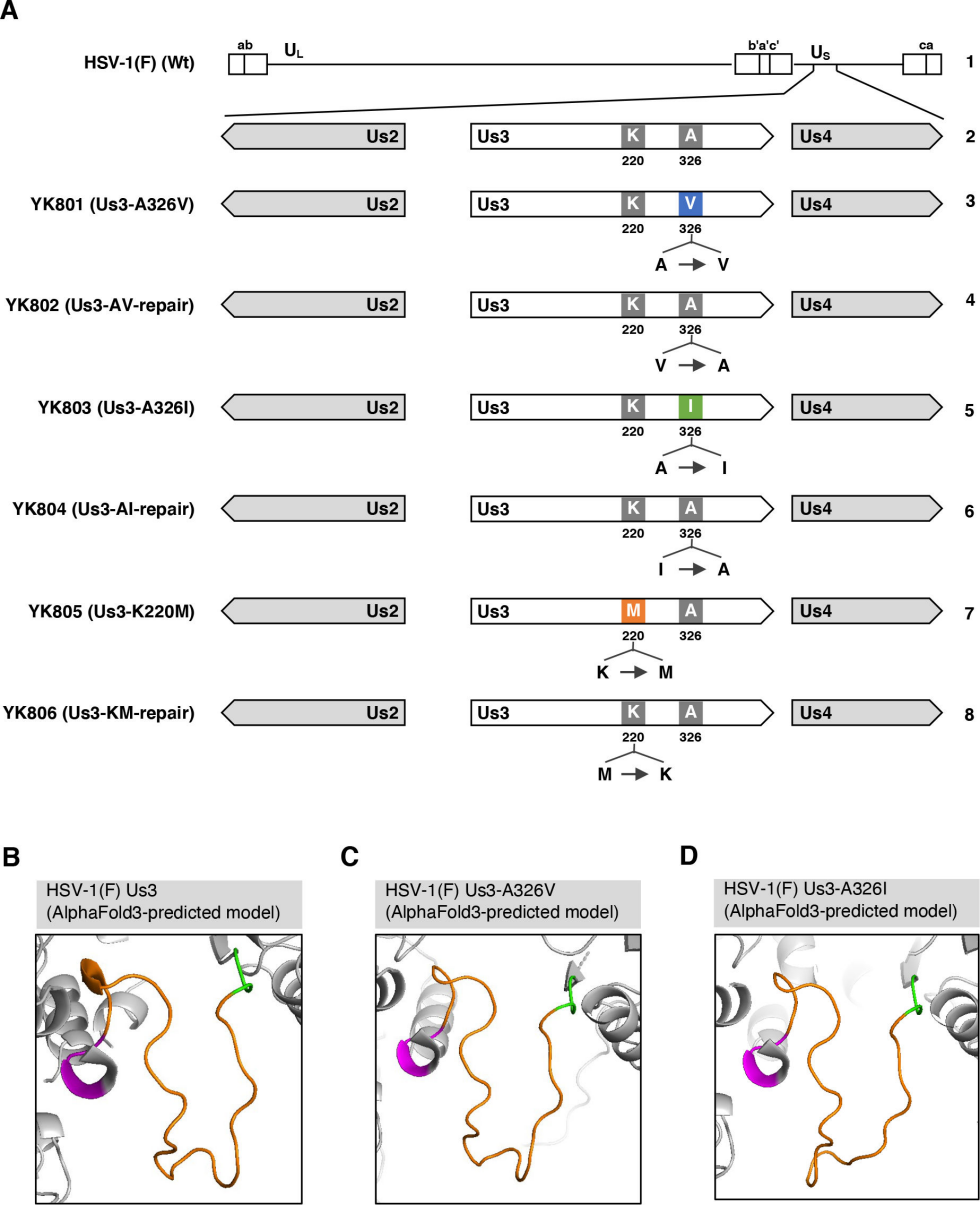
Genome structure of HSV-1(F) and the recombinant viruses used in this study and zoomed-in views of the A-loop regions in mutant Us3. (**A**) Line 1, wild-type HSV-1(F) genome; line 2, domains of Us2 to Us4; lines 3 to 8, recombinant viruses with mutations in Us3. (B to D) Zoomed-in views highlighting the A-loop regions of wild-type HSV-1(F) Us3 (**B**) and its mutants carrying A326V (**C**) and A326I (**D**) substitutions, as modeled by AlphaFold3.

To test our hypothesis, we followed a mutational analysis approach targeting Ala, as reported previously (55, 56), in which Ala was replaced with aliphatic hydrophobic structurally similar amino acids such as valine and isoleucine that exhibit modest increases in hydrophobicity and side-chain size compared with Ala. Specifically, we constructed recombinant viruses YK801 (Us3-A326V) and YK803 (Us3-A326I) (Fig. 2A), encoding mutant Us3 proteins where Ala-326 was replaced with valine (A326V) or isoleucine (A326I), respectively. AlphaFold3 structural modeling predicted that the amino acid substitutions at Us3 Ala-326 would not disrupt the formation of the A-loop in Us3 (Fig. 2B through D). Furthermore, the template modeling (TM) scores of Us3-A326V and Us3-A326I, which measure protein structural similarity (scores ≥ 0.5 suggest minimal changes to the overall protein folding) (57), were 0.68 and 0.79, respectively, compared with the wild-type Us3 structure. These results suggest that the amino acid substitutions at Us3 Ala-326 had a minimal effect on the structural characteristics of the A-loop and the global structure of Us3. As a control, we constructed a recombinant virus YK805 (Us3-K220M) (Fig. 2A) encoding a PK-dead mutant Us3 in which the lysine codon at position 220 (Lys-220) was replaced with methionine (K220M), as reported previously (37). In addition, we constructed repaired versions of these Us3 mutant viruses: YK802 (Us3-AV-repair), YK804 (Us3-AI-repair), and YK806 (Us3-KM-repair) (Fig. 2A).

### Effects of amino acid substitutions at Us3 Ala-326 on the phosphorylation levels of gB and Us3 in HSV-1-infected cells

**Fig 3:**
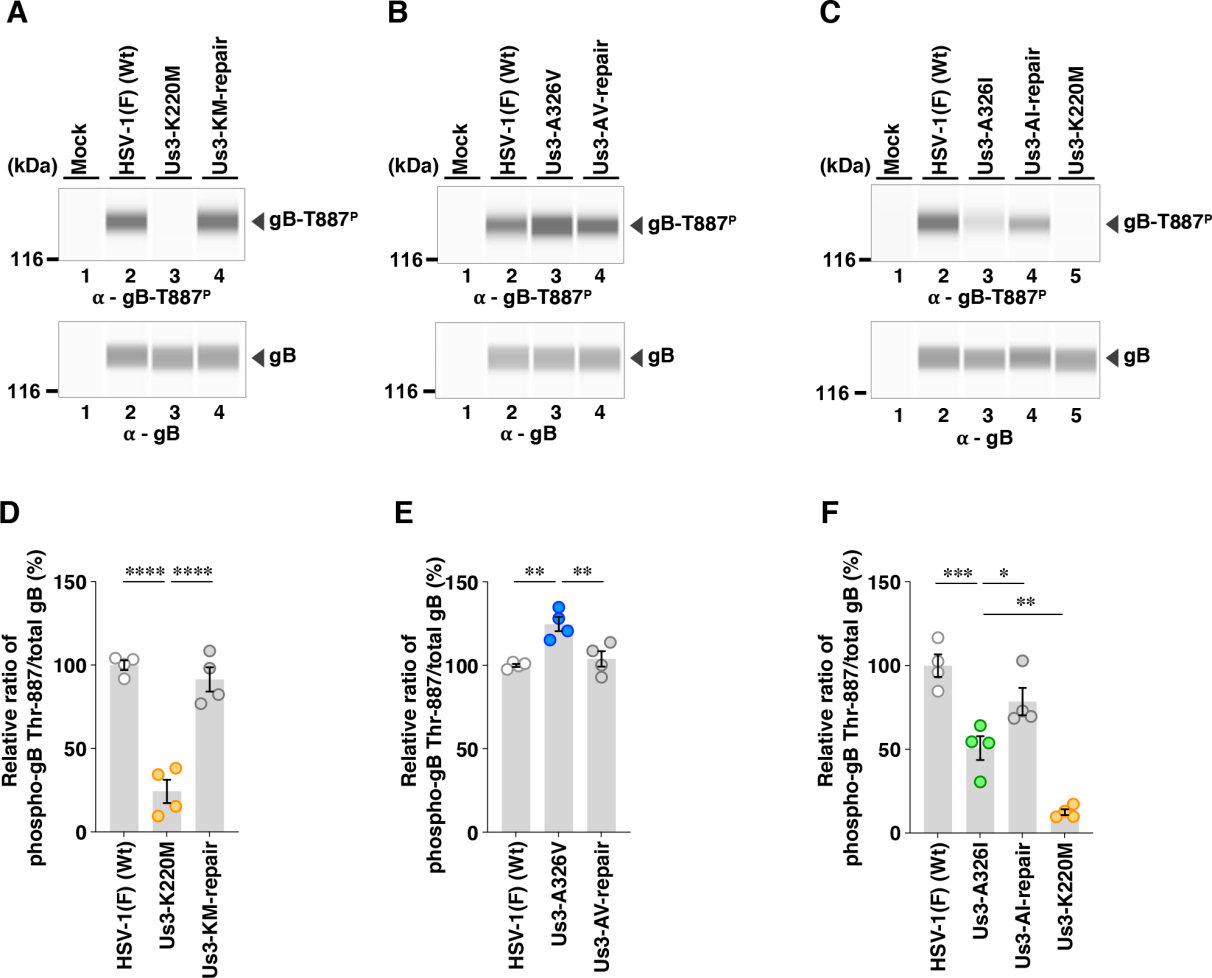
Effects of the A326V or A326I mutations in Us3 on the phosphorylation levels of gB Thr-887. (A to C) Vero cells were mock-infected or infected with wild-type HSV-1(F) (A to C), YK805 (Us3-K220M) (**A and C**), YK806 (Us3-KM-repair) (**A**), YK801 (Us3-A326V) (**B**), YK802 (Us3-AV-repair) (**B**), YK803 (Us3-A326I) (**C**), or YK804 (Us3-AI-repair) (**C**) at an MOI of 10, harvested 18 h post-infection and analyzed by immunoblotting using the SimpleWestern capillary system with antibodies to gB-T887^P^ or total gB. Digital images are representative of four independent experiments. A molecular mass marker is indicated on the left. (D to F) The accumulation levels of gB-T887^P^ in the experiments shown in panels A to C were quantified and normalized to those of total gB. Values are expressed as the mean ± standard error of the mean (SEM) of four independent experiments, relative to the values obtained from Vero cells infected with HSV-1(F), which were normalized to 100%. Statistical significance was assessed by one-way ANOVA followed by Tukey’s test. ^*^; *P*  <  0.05, ^**^; *P*  <  0.01, ^***^; *P*  <  0.001, ^****^; *P*  <  0.0001, n.s., not significant.

To examine the effects of the A326V and A326I mutations in Us3 on the phosphorylation levels of its substrates in infected cells, we utilized phospho-specific monoclonal antibodies against Us3 substrates that we had previously developed (24, 29). These antibodies specifically recognize gB phosphorylated at Thr-887 (gB-T887^P^) and Us3 phosphorylated at Ser-147 (Us3-S147^P^) (24, 29). Simian kidney epithelial Vero cells were mock-infected or infected with wild-type HSV-1(F), YK801 (Us3-A326V), YK802 (Us3-AV-repair), YK803 (Us3-A326I), YK804 (Us3-AI-repair), YK805 (Us3-K220M), or YK806 (Us3-KM-repair) at multiplicities of infection (MOI) of 10 and were harvested at 18 h post-infection. To detect the phosphorylated gB and Us3 using these phospho-specific antibodies, we used the SimpleWestern capillary system (58, 59), which provides the highly linear quantification of target proteins. In agreement with our previous studies using conventional immunoblotting (24, 29), the accumulation levels of gB-T887^P^, detected as a 145 kDa protein in this system, were markedly diminished in lysates of cells infected with YK805 (Us3-K220M) encoding the PK-dead Us3, compared with those in lysates of cells infected with wild-type HSV-1(F) or YK806 (Us3-KM-repair) (Fig. 3A and D).

**Fig 4:**
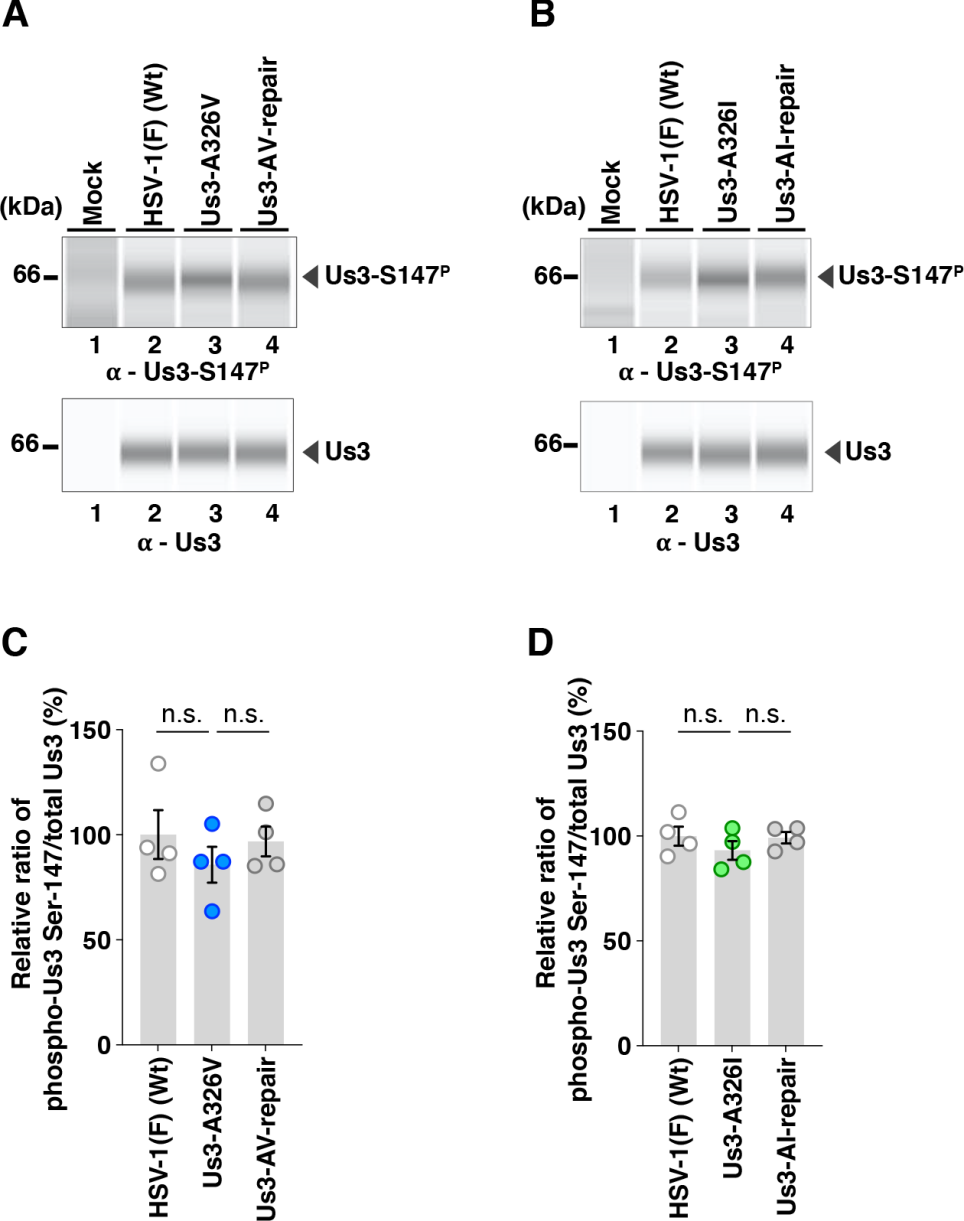
Effects of the A326V or A326I mutations in Us3 on the phosphorylation level of Us3 Ser-147 itself. (**A and B**) Vero cells were mock-infected or infected with wild-type HSV-1(F) (**A and B**), YK801 (Us3-A326V) (**A**), YK802 (Us3-AV-repair) (**A**), YK803 (Us3-A326I) (**B**), or YK804 (Us3-AI-repair) (**B**) at an MOI of 10, harvested 18 h post-infection, and analyzed by immunoblotting using the SimpleWestern capillary system with antibodies to Us3-S147^P^ or total Us3. Digital images are representative of four independent experiments. A molecular mass marker is indicated on the left. (**C and D**) The accumulation levels of Us3-S147^P^ in the experiments shown in panels **A and B** were quantified and normalized to those of total Us3. Values are expressed as the mean ± SEM of four independent experiments, relative to the values obtained from Vero cells infected with HSV-1(F), which were normalized to 100%. Statistical significance was assessed by one-way ANOVA followed by Tukey’s test. n.s., not significant.

The A326V mutation in Us3 significantly increased the accumulation levels of gB-T887^P^ (Fig. 3B and E). In contrast, the A326I mutation in Us3 significantly decreased the accumulation levels of gB-T887^P^, although these levels remained significantly higher than those observed with the PK-dead mutation in Us3 (Fig. 3C and F). The A326V and A326I mutations had little effect on the overall accumulation levels of Us3-S147^P^ (Fig. 4). None of the Us3 mutations had an obvious effect on the accumulation levels of total gB and Us3 proteins (Fig. 3A through C, 4A and B). These results indicated that Us3 Ala-326 was required for the proper level of phosphorylation at gB Thr-887, but not at Us3 Ser-147, in HSV-1-infected cells.

### Effects of amino acid substitutions at Us3 Ala-326 on the phosphorylation levels of Us3 targets recognized by anti-phospho-PKA and anti-phospho-Akt substrate antibodies in HSV-1-infected cells

**Fig 5:**
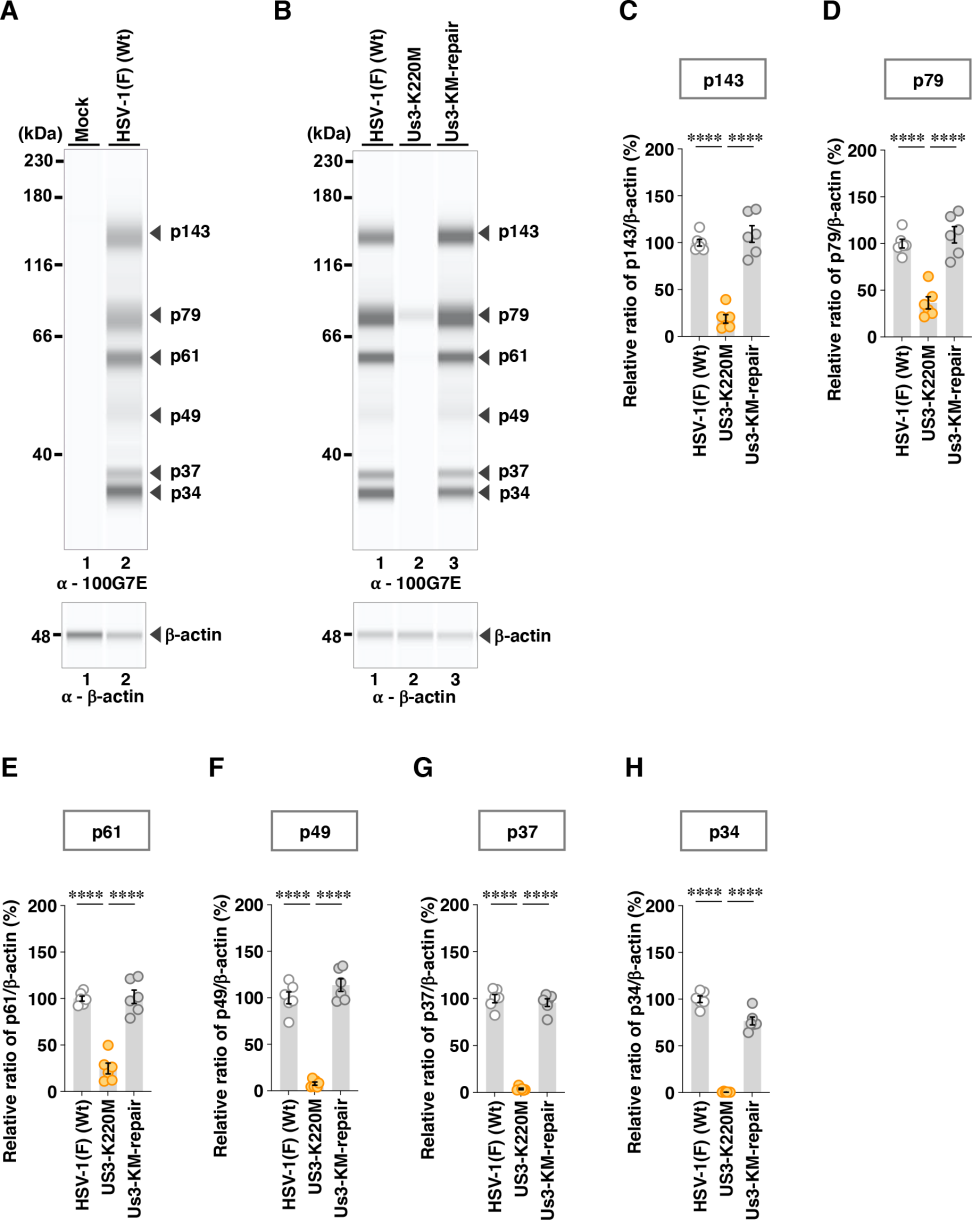
Effects of the PK-dead mutations in Us3 on the phosphorylation levels of proteins detected by the 100G7E antibody. (**A and B**) Vero cells were mock-infected (**A**) or infected with wild-type HSV-1(F) (**A and B**), YK805 (Us3-K220M) (**B**), or YK806 (Us3-KM-repair) (**B**) at an MOI of 10, harvested 18 h post-infection, and analyzed by immunoblotting using the SimpleWestern capillary system with antibodies to anti-phospho-PKA substrate 110G7E or β-actin. Digital images are representative of three (**A**) or six (**B**) independent experiments. A molecular mass marker is indicated on the left. (C to H) The accumulation levels corresponding to p143 (**C**), p79 (**D**), p61 (**E**), p49 (**F**), p37 (**G**), and p34 (**H**) in the experiments shown in (**B**) were quantified and normalized to those of β-actin. Values represent the mean ± SEM of six independent experiments, expressed relative to the values obtained from Vero cells infected with HSV-1(F), which were normalized to 100%. Statistical significance was assessed by one-way ANOVA followed by Tukey’s test: ^****^; *P* <  0.0001, n.s., not significant.

**Fig 6:**
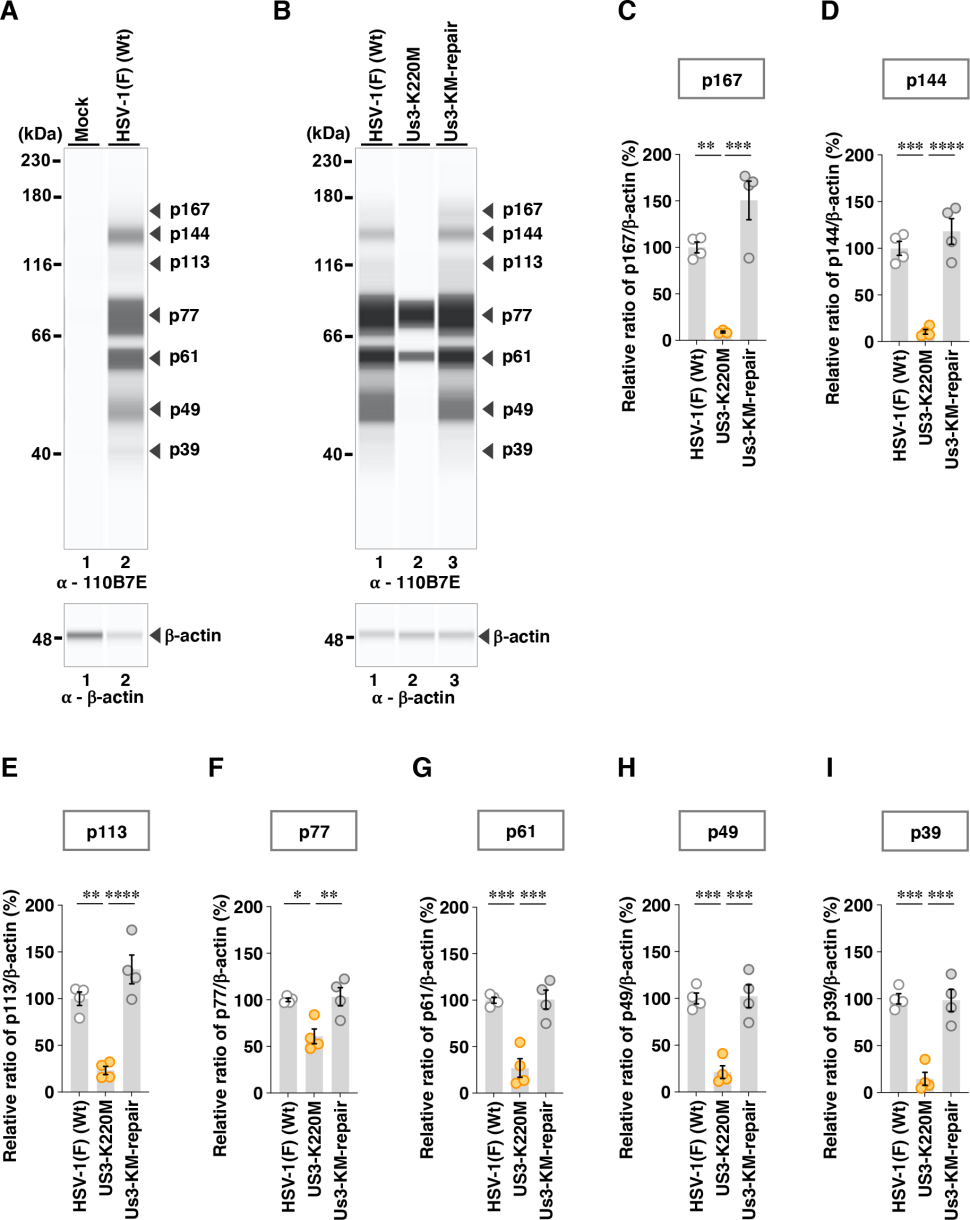
Effects of the PK-dead mutations in Us3 on the phosphorylation levels of proteins detected by the 110B7E antibody. (**A and B**) Vero cells were mock-infected (**A**) or infected with wild-type HSV-1(F) (**A and B**), YK805 (Us3-K220M) (**B**), or YK806 (Us3-KM-repair) (**B**) at an MOI of 10, harvested 18 h post-infection, and analyzed by immunoblotting using the SimpleWestern capillary system with antibodies to anti-phospho-AKT substrate 110B7E or β-actin. Digital images are representative of three (**A**) or four (**B**) independent experiments. A molecular mass marker is indicated on the left. (C to I) The accumulation levels corresponding to p167 (**C**), p144 (**D**), p113 (**E**), p77 (**F**), p61 (**G**), p49 (**H**), and p39 (**I**) in the experiments shown in (**B**) were quantified and normalized to those of β-actin. Values represent the mean ± SEM of four independent experiments, expressed relative to the values obtained from Vero cells infected with HSV-1(F), which were normalized to 100%. Statistical significance was assessed by one-way ANOVA followed by Tukey’s test: ^*^; *P*  <  0.05, ^**^; *P*  <  0.01, ^***^; *P*  <  0.001, ^****^; *P*  <  0.0001, n.s., not significant.

**Fig 7:**
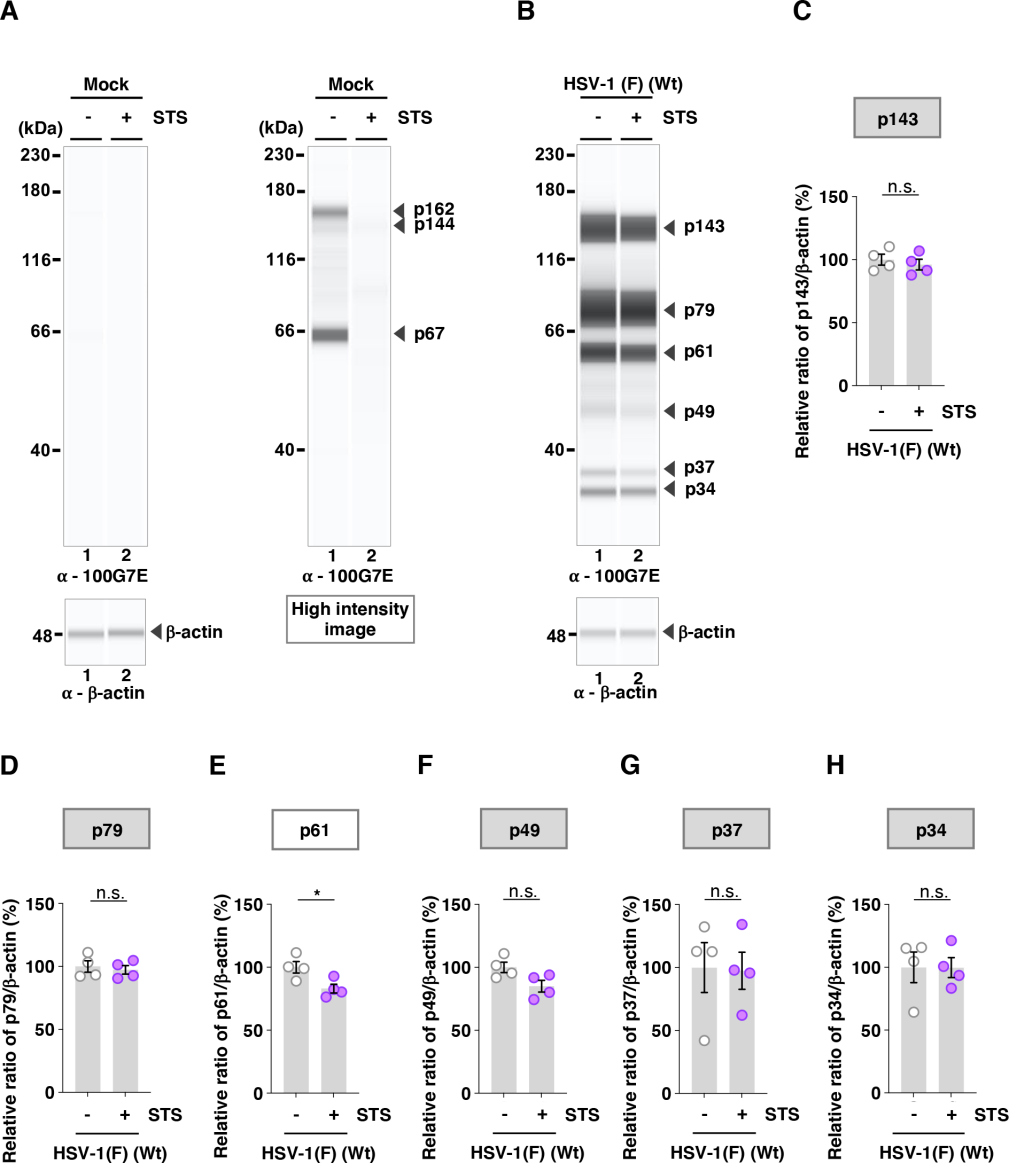
Effects of the PKA inhibitor on the phosphorylation levels of proteins detected by the 100G7E antibody. (**A and B**) Vero cells were mock-infected (**A**) or infected with wild-type HSV-1(F) (**B**) at an MOI of 10, treated with 2 µM staurosporine (STS) or DMSO 12 h post-infection, harvested 18 h post-infection, and analyzed by immunoblotting using the SimpleWestern capillary system with antibodies to anti-phospho-PKA substrate 100G7E or β-actin. Digital images are representative of three (**A**) or four (**B**) independent experiments. A molecular mass marker is indicated on the left. A high-intensity image is also shown (right panel of A). (C to H) The accumulation levels corresponding to p143 (**C**), p79 (**D**), p61 (**E**), p49 (**F**), p37 (**G**), and p34 (**H**) in the experiments shown in panel **B** were quantified and normalized to those of β-actin. Values represent the mean ± SEM of four independent experiments, expressed relative to the values obtained from DMSO-treated HSV-1(F)-infected Vero cells, which were normalized to 100%. Statistical significance was assessed by an unpaired two-tailed Student *t*-test: ^*^; *P*  <  0.05, n.s., not significant.

To further investigate the effects of the A326V and A326I mutations in Us3 on the phosphorylation levels of other Us3 targets in infected cells, we utilized two additional phospho-specific antibodies: anti-phospho-PKA substrate 100G7E and anti-phospho-AKT substrate 110B7E. These antibodies broadly recognize proteins containing phosphorylated serine or threonine residues flanked by arginine at positions −3 and −2 (RRXS or RRXT), or at position −3 (RXXS or RXXT), respectively, which closely resembles the substrate motif targeted by Us3 (17, 18). Indeed, it was reported that antibodies can detect Us3-dependent phosphorylation on Us3 substrates (17, 21, 60, 61). Vero cells were mock-infected or infected with wild-type HSV-1(F), YK801 (Us3-A326V), YK802 (Us3-AV-repair), YK803 (Us3-A326I), YK804 (Us3-AI-repair), YK805 (Us3-K220M), or YK806 (Us3-KM-repair) at an MOI of 10. Then, cells were harvested at 18 h post-infection and analyzed using the SimpleWestern capillary system. Although the anti-phospho-PKA substrate 100G7E and anti-phospho-AKT substrate 110B7E antibodies exhibited very weak reactivity against lysates from mock-infected cells, resulting in minimal protein detection, they clearly detected multiple proteins in lysates from cells infected with wild-type HSV-1(F) (Fig. 5A and 6A). Specifically, the anti-phospho-PKA substrate 100G7E antibody detected six proteins with estimated individual molecular weights of 143, 79, 61, 49, 37, and 34 kDa (p143, p79, p61, p49, p37, and p34) (Fig. 5A), whereas the anti-phospho-AKT substrate 110B7E antibody detected seven proteins with estimated individual molecular weights of 167, 144, 113, 77, 61, 49, and 39 kDa (p167, p144, p113, p77, p61, p49, and p39) (Fig. 6A). The accumulation levels of all these proteins in lysates of cells infected with YK805 (Us3-K220M) were significantly decreased compared with those in lysates of cells infected with wild-type HSV-1(F) or YK806 (Us3-KM-repair) (Fig. 5B through H and 6B through I). Since Us3 has previously been reported to mediate post-translational modification of PKA in HSV-1-infected cells (17), we tested the effect of treatment with staurosporine (STS), a PKA inhibitor, on the accumulation levels of proteins detected by the anti-phospho-PKA substrate 100G7E antibody. In mock-infected Vero cells, STS treatment abolished all bands detected by the anti-phospho-PKA substrate 100G7E antibody (Fig. 7A). In contrast, in HSV-1(F)-infected Vero cells, STS treatment affected only one of six phospho-protein bands (Fig. 7B through H): the p61 band was reduced by 16.9% (Fig. 7E), whereas the Us3-kinase-dead mutant showed a 75.1% reduction (Fig. 5E). Collectively, these results suggested that Us3 predominantly mediated the phosphorylation of these proteins in HSV-1-infected cells in a PKA-independent manner.

**Fig 8:**
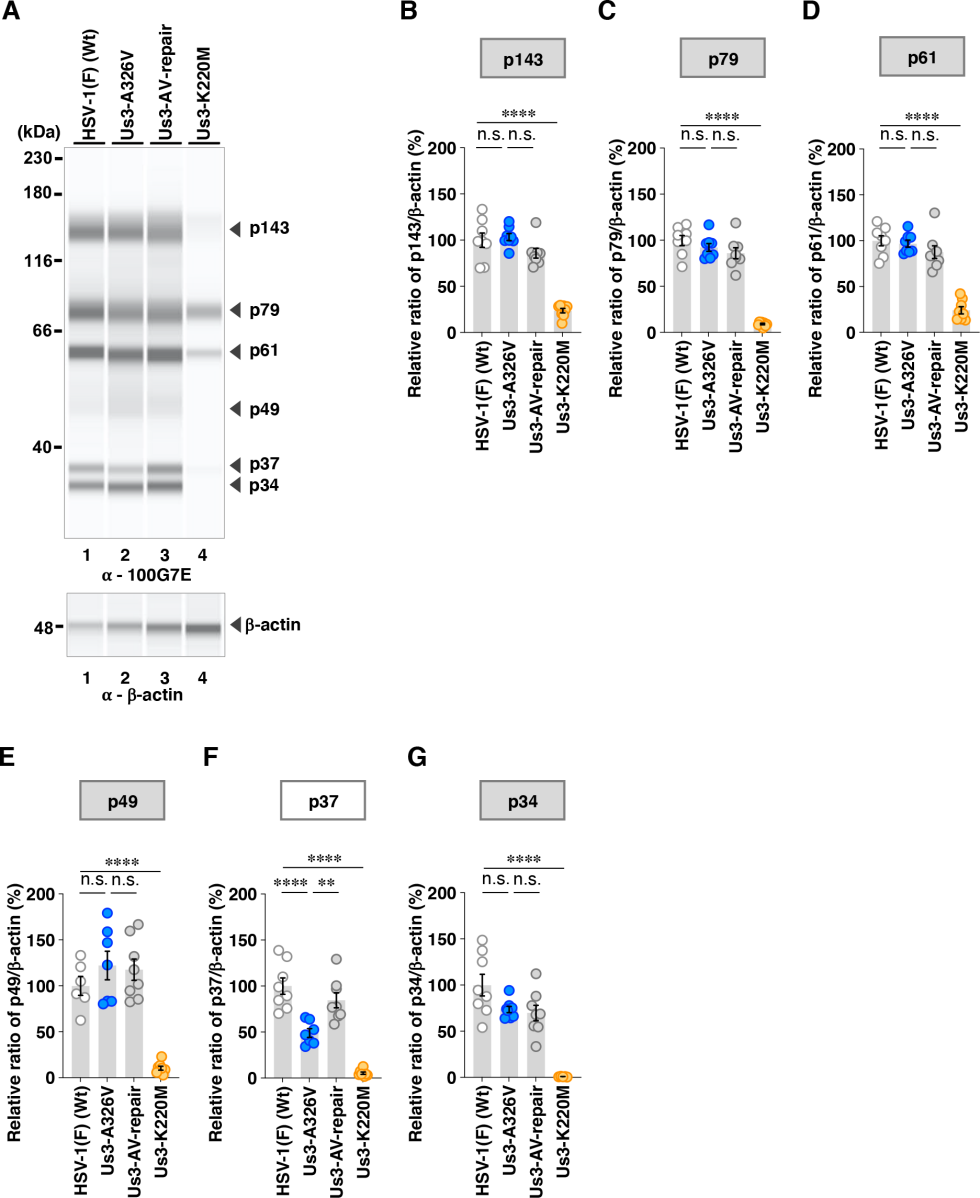
Effects of the A326V mutations in Us3 on the phosphorylation levels of proteins detected by the 100G7E antibody. (**A**) Vero cells were infected with wild-type HSV-1(F), YK801 (Us3-A326V), YK802 (Us3-AV-repair), or YK805 (Us3-K220M) at an MOI of 10, harvested 18 h post-infection, and analyzed as in Fig. 5A. Digital images are representative of eight independent experiments. A molecular mass marker is indicated on the left. (B to G) The accumulation levels corresponding to p143 (**B**), p79 (**C**), p61 (**D**), p49 (**E**), p37 (**F**), and p34 (**G**) in the experiments shown in panel **A** were quantified and normalized to those of β-actin. Values represent the mean ± SEM of eight independent experiments, expressed relative to the values obtained from Vero cells infected with HSV-1(F), which were normalized to 100%. Statistical significance was assessed by one-way ANOVA followed by Tukey’s test: ^**^; *P* <  0.01, ^****^; *P*  <  0.0001, n.s., not significant.

**Fig 9:**
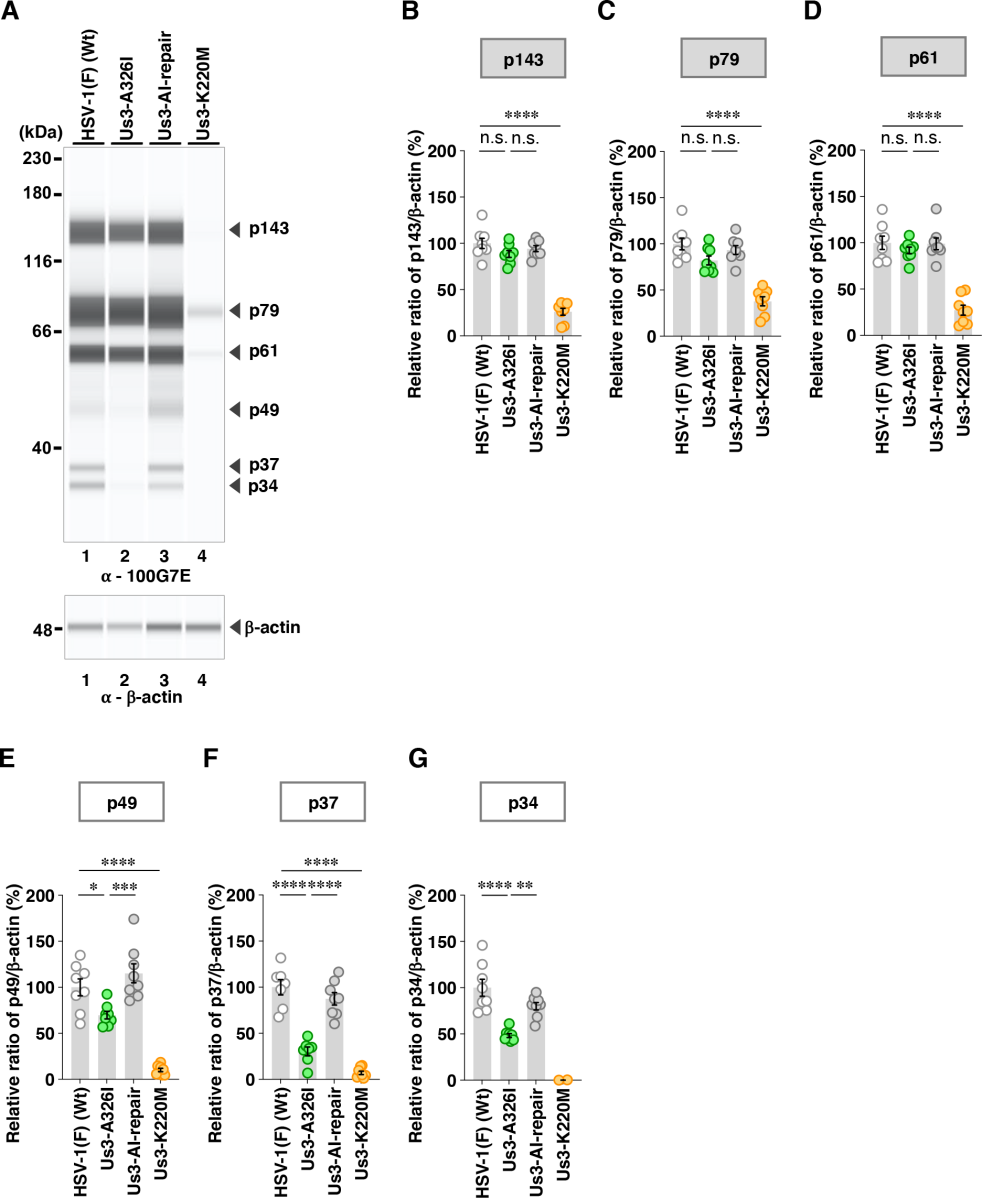
Effects of the A326I mutations in Us3 on the phosphorylation levels of proteins detected by the 100G7E antibody. (**A**) Vero cells were mock-infected or infected with wild-type HSV-1(F), YK803 (Us3-A326I), YK805 (Us3-K220M), or YK804 (Us3-AI-repair) at an MOI of 10, harvested 18 h post-infection, and analyzed as in Fig. 5A. Digital images are representative of eight independent experiments. A molecular mass marker is indicated on the left. (B to G) The accumulation levels corresponding to p143 (**B**), p79 (**C**), p61 (**D**), p49 (**E**), p37 (**F**), and p34 (**G**) in the experiments shown in panel **A** were quantified and normalized to those of β-actin. Values represent the mean ± SEM of eight independent experiments, expressed relative to the values obtained from Vero cells infected with HSV-1(F), which were normalized to 100%. Statistical significance was assessed by one-way ANOVA followed by Tukey’s test: ^*^; *P*  <  0.05, ^**^; *P*  <  0.01, ^***^; *P*  <  0.001, ^****^; *P*  <  0.0001, n.s., not significant.

Among the proteins detected by the anti-phospho-PKA substrate 100G7E antibody, the A326V and A326I mutations in Us3 significantly decreased the accumulation levels of p37 (Fig. 8 and 9). In addition to the decrease in p37 (Fig. 9), the A326I mutation in Us3 significantly reduced the accumulation levels of p49 and p34, whereas the A326V mutation in Us3 had little effect on these protein levels (Fig. 8).

**Fig 10:**
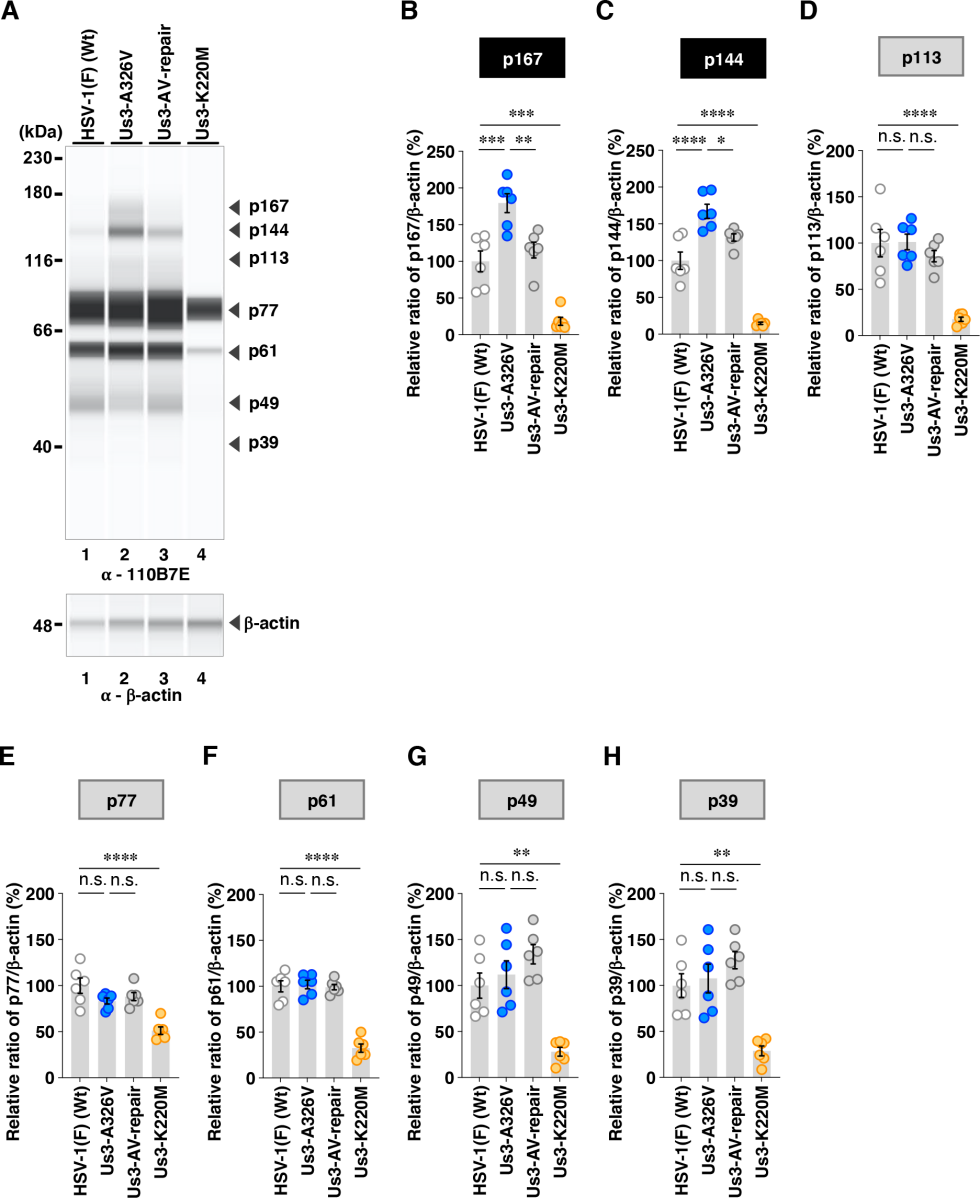
Effects of the A326V mutations in Us3 on the phosphorylation levels of proteins detected by the 110B7E antibody. (**A**) Vero cells were infected with wild-type HSV-1(F), YK801 (Us3-A326V), YK802 (Us3-AV-repair), or YK805 (Us3-K220M) at an MOI of 10, harvested 18 h post-infection, and analyzed as in Fig. 6A. Digital images are representative of six independent experiments. A molecular mass marker is indicated on the left. (B to H) The accumulation levels corresponding to p167 (**B**), p144 (**C**), p113 (**D**), p77 (**E**), p61 (**F**), p49 (**G**), and p39 (**H**) in the experiments shown in panel **A** were quantified and normalized to those of β-actin. Values represent the mean ± SEM of six independent experiments, expressed relative to the values obtained from Vero cells infected with HSV-1(F), which were normalized to 100%. Statistical significance was assessed by one-way ANOVA followed by Tukey’s test: ^*^; *P*  <  0.05, ^**^; *P*  <  0.01, ^***^; *P*  <  0.001, ^****^; *P*  <  0.0001, n.s., not significant.

**Fig 11:**
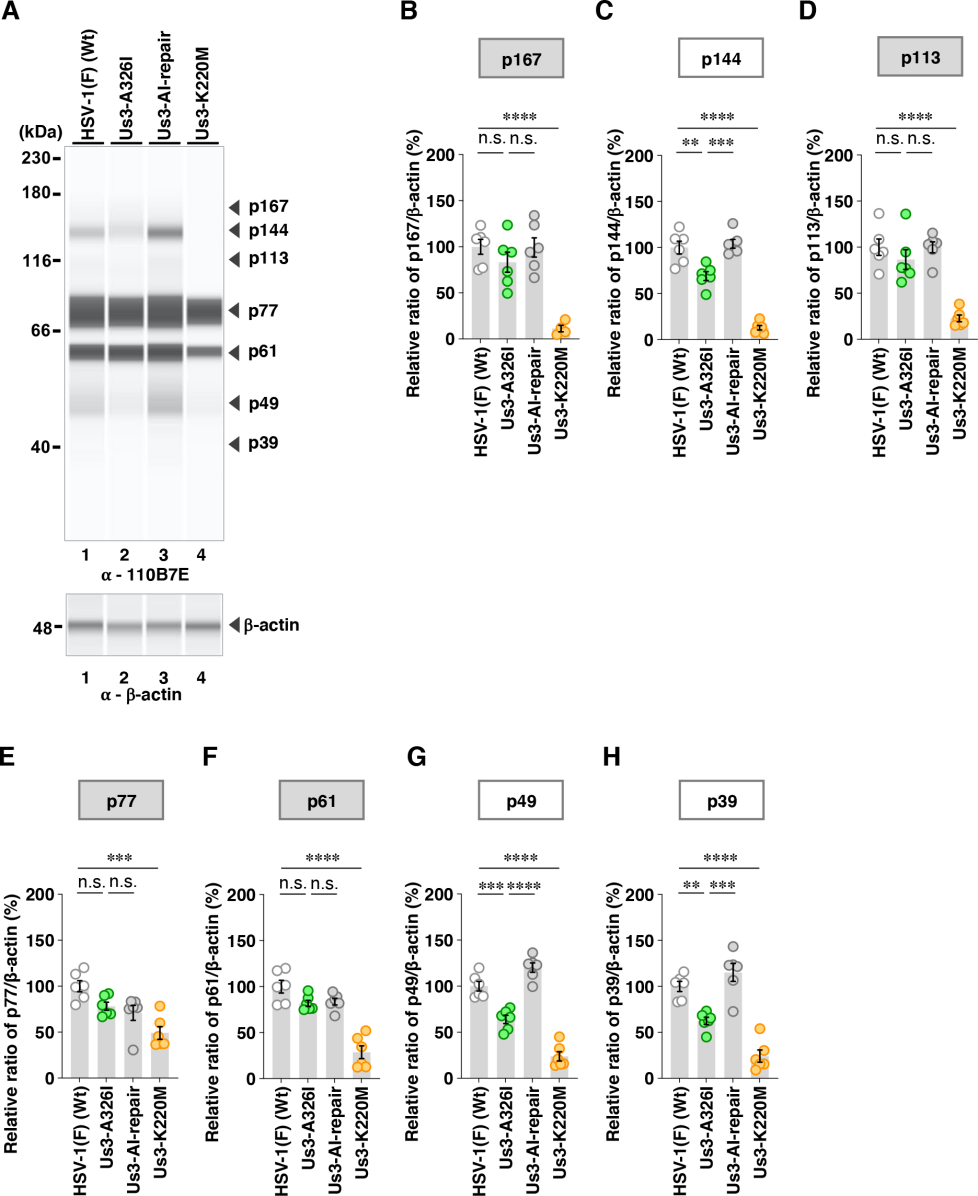
Effects of the A326I mutations in Us3 on the phosphorylation levels of proteins detected by the 110B7E antibody. (**A**) Vero cells were infected with wild-type HSV-1(F), YK803 (Us3-A326I), YK804 (Us3-AI-repair), or YK805 (Us3-K220M) at an MOI of 10, harvested 18 h post-infection and analyzed as in Fig. 6A. Digital images are representative of six independent experiments. A molecular mass marker is indicated on the left. (B to H) The accumulation levels corresponding to p167 (**B**), p144 (**C**), p113 (**D**), p77 (**E**), p61 (**F**), p49 (**G**), and p39 (**H**) in the experiments shown in panel **A** were quantified and normalized to those of β-actin. Values represent the mean ± SEM of six independent experiments, expressed relative to the values obtained from Vero cells infected with HSV-1(F), which were normalized to 100%. Statistical significance was assessed by one-way ANOVA followed by Tukey’s test: ^*^; *P* <  0.05, ^**^; *P* <  0.01, ^***^; *P*  <  0.001, ^****^; *P*  <  0.0001, n.s., not significant.

Among the proteins detected by the anti-phospho-Akt substrate 110B7E antibody, the A326V mutation in Us3 significantly increased the accumulation levels of p167 and p144 (Fig. 10). In contrast, the A326I mutation in Us3 significantly decreased the accumulation levels of p144 but had little effect on p167 (Fig. 11). Furthermore, the A326I mutation in Us3 significantly reduced the accumulation levels of p49 and p39 (Fig. 11).

Of note, the effects of the two mutations on p49, detected by the anti-phospho-PKA substrate 100G7E antibody, were comparable with the effects on p49 detected by the anti-phospho-AKT substrate 110B7E antibody (Fig. 8 to 11). These results suggested that p49 detected by the anti-phospho-PKA substrate 100G7E antibody was the same protein as p49 detected by the anti-phospho-AKT substrate 110B7E antibody.

**Fig 12:**
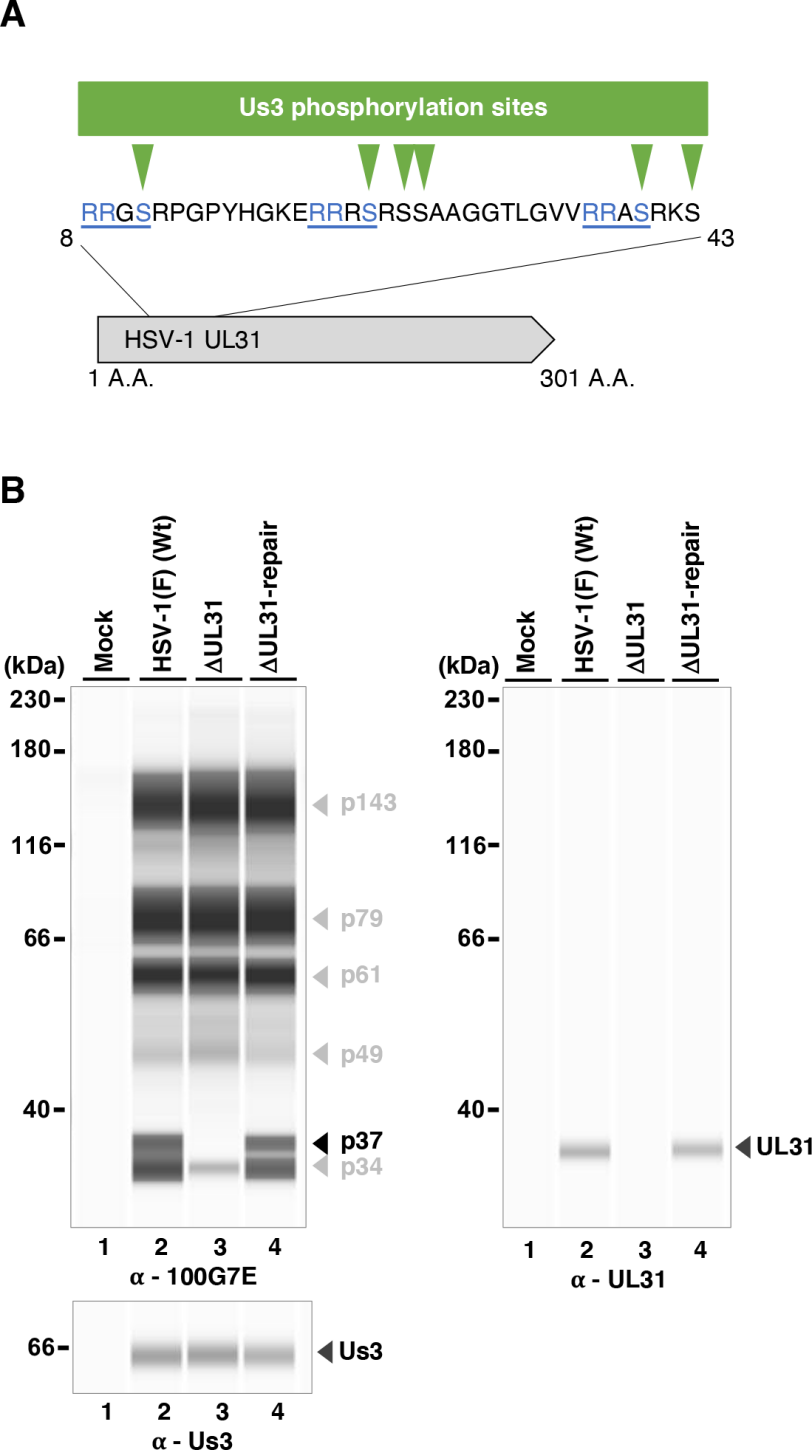
Effects of the null mutation in UL31 on the phosphorylation levels of proteins detected by the 100G7E antibody. (**A**) Schematic of the N-terminal region of HSV-1 UL31 (aa 8-43). Blue underlining marks the sequence matching the 100G7E antibody epitope; green arrowheads denote reported Us3 phosphorylation sites. The corresponding region within full-length UL31 (aa 1-301) is shown below. (**B**) Vero cells were mock-infected or infected with wild-type HSV-1(F), YK720 (ΔUL31), or YK721 (ΔUL31-repair) at an MOI of 10, harvested 18 h post-infection, and analyzed by immunoblotting using the SimpleWestern capillary system with antibodies to anti-phospho-PKA substrate 100G7E, UL31 or Us3. Digital images are representative of three independent experiments. A molecular mass marker is indicated on the left.

We also attempted to identify viral protein(s) showing differential phosphorylation upon the mutation(s) of Us3 Ala-326 by using null-mutant viruses of various viral substrates of Us3. Among the Us3 substrate-null mutant viruses tested, the UL31-null mutation completely abolished the p37 band detected by the anti-phospho-PKA-substrate 100G7E antibody (100G7E) (Fig. 12). In addition, the anti-UL31 antibody reacted with a band of molecular weight comparable with p37 in lysates of HSV-1(F)-infected cells in the SimpleWestern capillary system (Fig. 12). These results suggested that p37 corresponded to UL31, a known substrate of Us3 (32, 47).

These findings indicated that Us3 Ala-326 was required to achieve the proper levels of phosphorylation of at least six proteins mediated by Us3, including p34, p37 (UL31), p39, p49, p144, and p167, in addition to gB; however, it was not required for the phosphorylation of other proteins mediated by Us3 including p61, p71, p79, p113, and p143 in HSV-1-infected cells, suggesting that this residue was required for the proper fine-tuning of Us3-mediated phosphorylation across its target repertoire in HSV-1-infected cells.

### Effects of amino acid substitutions at Us3 Ala-326 on viral growth kinetics and plaque size in cell cultures

**Fig 13:**
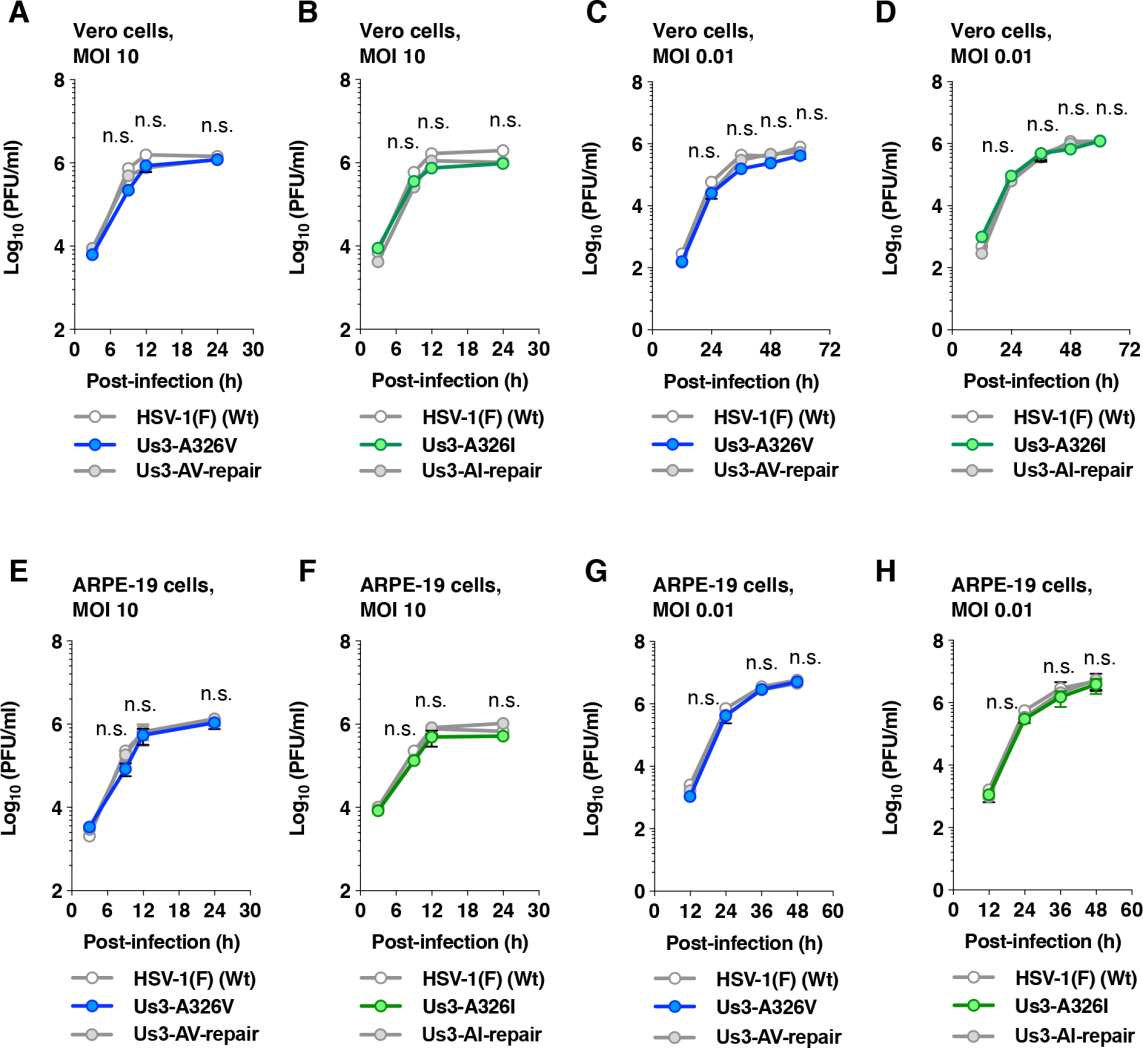
Effects of the A326V or A326I mutations in Us3 on viral replication in cell cultures. (A to H) Vero (A to D) or ARPE-19 (E to H) cells were infected with wild-type HSV-1(F) (A to H), YK801 (Us3-A326V) (A, C, E, and G), YK802 (Us3-AV-repair) (A, C, E, and G), YK803 (Us3-A326I) (B, D, F, and H), or YK804 (Us3-AI-repair) (B, D, F, and H) at an MOI of 10 (A, B, E, and F) or 0.01 (C, D, G, and H). Total virus from the cell culture supernatants and infected cells was harvested at the indicated times and assayed on Vero cells. Each data point represents the mean ± SEM of the results of three (**C and D**), four (**A and B**), five (**G and H**), or six (**E and F**) independent experiments. Statistical significance was assessed by one-way ANOVA followed by Tukey’s test. The *P*-values for comparisons between Us3-A326V (A, C, E, and G), Us3-A326I (B, D, F, and H), and their corresponding repair viruses are indicated. n.s., not significant.

To examine the effects of the A326V and A326I mutations in Us3 on HSV-1 infection in cell cultures, we analyzed the growth kinetics and plaque size of Vero and human retinal pigment epithelial ARPE-19 cells. Vero and ARPE-19 cells infected with wild-type HSV-1(F), YK801 (Us3-A326V), YK802 (Us3-AV-repair), YK803 (Us3-A326I), or YK804 (Us3-AI-repair) at MOIs of 10 or 0.01 were harvested at various times post-infection, and total virus titers in the infected cells and cell culture supernatants were assayed. The viral growth curves of YK801 (Us3-A326V) and YK803 (Us3-A326I) were similar to those of wild-type HSV-1(F) and their repaired viruses in Vero and ARPE-19 cells at MOIs of 10 and 0.01 (Fig. 13).

**Fig 14:**
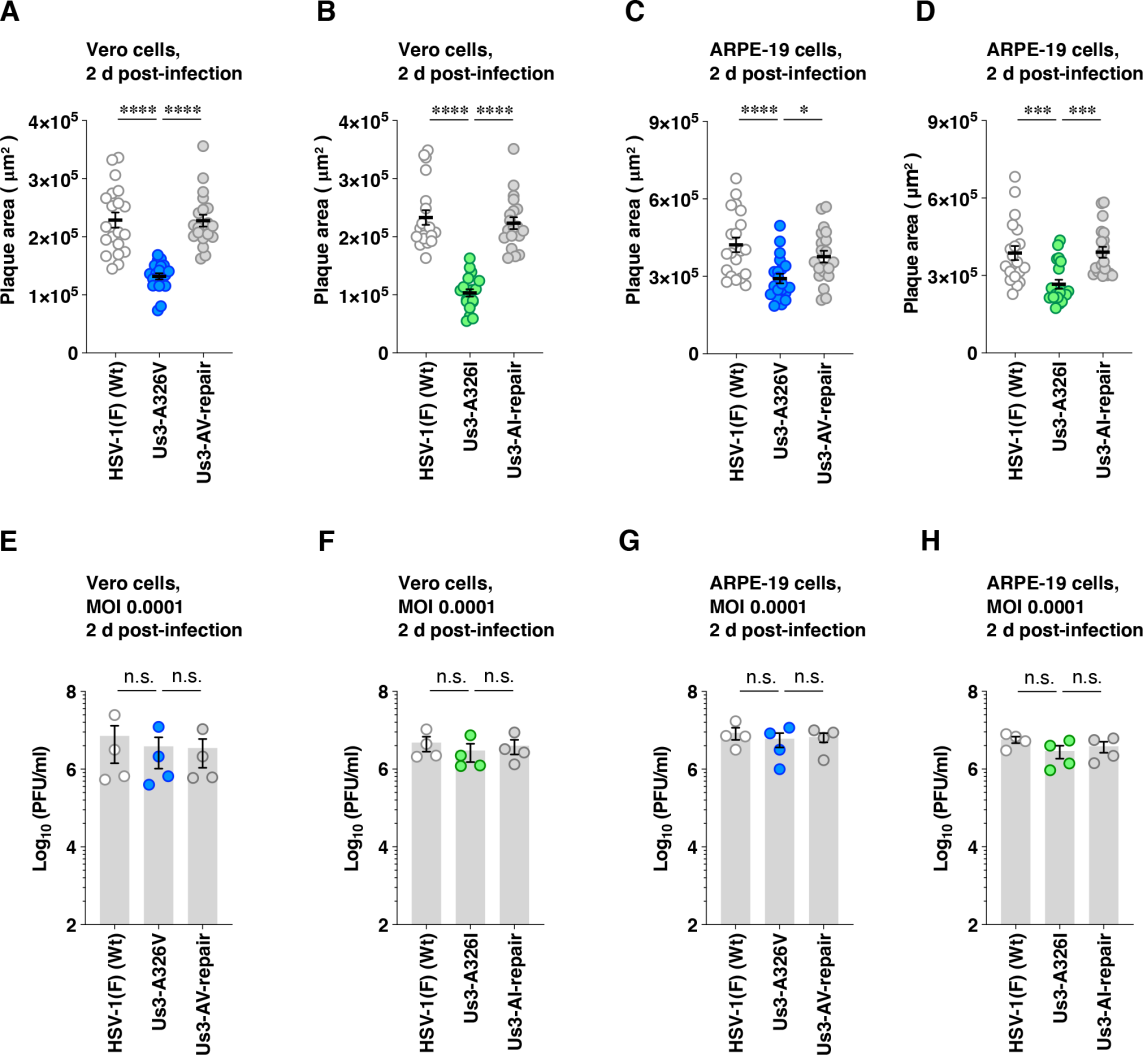
Effects of the A326V or A326I mutations in Us3 on cell-cell viral spread in cell cultures. (A to D) Vero (**A and B**) or ARPE-19 (**C and D**) cells were infected with wild-type HSV-1(F) (A to D), YK801 (Us3-A326V) (**A and C**), YK802 (Us3-AV-repair) (**A and C**), YK803 (Us3-A326I) (**B and D**), or YK804 (Us3-AI-repair) (**B and D**) under plaque assay conditions (at an MOI of 0.0001). The diameters of 20 single plaques for each of the indicated viruses were measured 2 days post-infection. Each point represents a single plaque area. The horizontal line and error bars indicate the mean ± SEM for each group. (E to H) Vero cells (**E and F**) or ARPE-19 (**G and H**) cells were infected with wild-type HSV-1(F) (E to H), YK801 (Us3-A326V) (**E and G**), YK802 (Us3-AV-repair) (**E and G**), YK803 (Us3-A326I) (**F and H**), or YK804 (Us3-AI-repair) (**F and H**) at an MOI of 0.0001 as in panels A to D. Total virus from the cell culture supernatants and infected cells was harvested 2 days post-infection and assayed on Vero cells. Each data point represents the mean ± SEM of the results of four independent experiments (E to H). Statistical significance was assessed by one-way ANOVA followed by Tukey’s test. ^*^; *P*  <  0.05, ^***^; *P*  <  0.001, ^****^; *P*  <  0.0001, n.s., not significant.

Next, Vero and ARPE-19 cells were infected with wild-type HSV-1(F), YK801 (Us3-A326V), YK802 (Us3-AV-repair), YK803 (Us3-A326I), or YK804 (Us3-AI-repair) at an MOI of 0.0001 under plaque assay conditions. At 2 days post-infection, the plaque size was measured. YK801 (Us3-A326V) and YK803 (Us3-A326I) produced significantly smaller plaques than wild-type HSV-1(F), YK802 (Us3-AV-repair), and YK804 (Us3-AI-repair) in Vero and ARPE-19 cells (Fig. 14A through D). Of note, progeny virus yields of Vero or APRE-19 cells infected with YK801 (Us3-A326V) or YK803 (Us3-A326I) at an MOI of 0.0001 at 2 d post-infection were similar to those of cells infected with wild-type HSV-1(F) or each of their repaired viruses (Fig. 14E through H). This eliminated the possibility that the reduced plaque sizes observed with the A326V and A326I mutations in Us3 were due to impaired viral replication under the plaque assay conditions, including an MOI of 0.0001 and a 2-day infection period. Thus, Us3 Ala-326 was required for efficient HSV-1 cell-to-cell spread without affecting viral replication in these cells.

### Effects of amino acid substitutions at Us3 Ala-326 on HSV-1 replication and pathogenicity in mice

**Fig 15:**
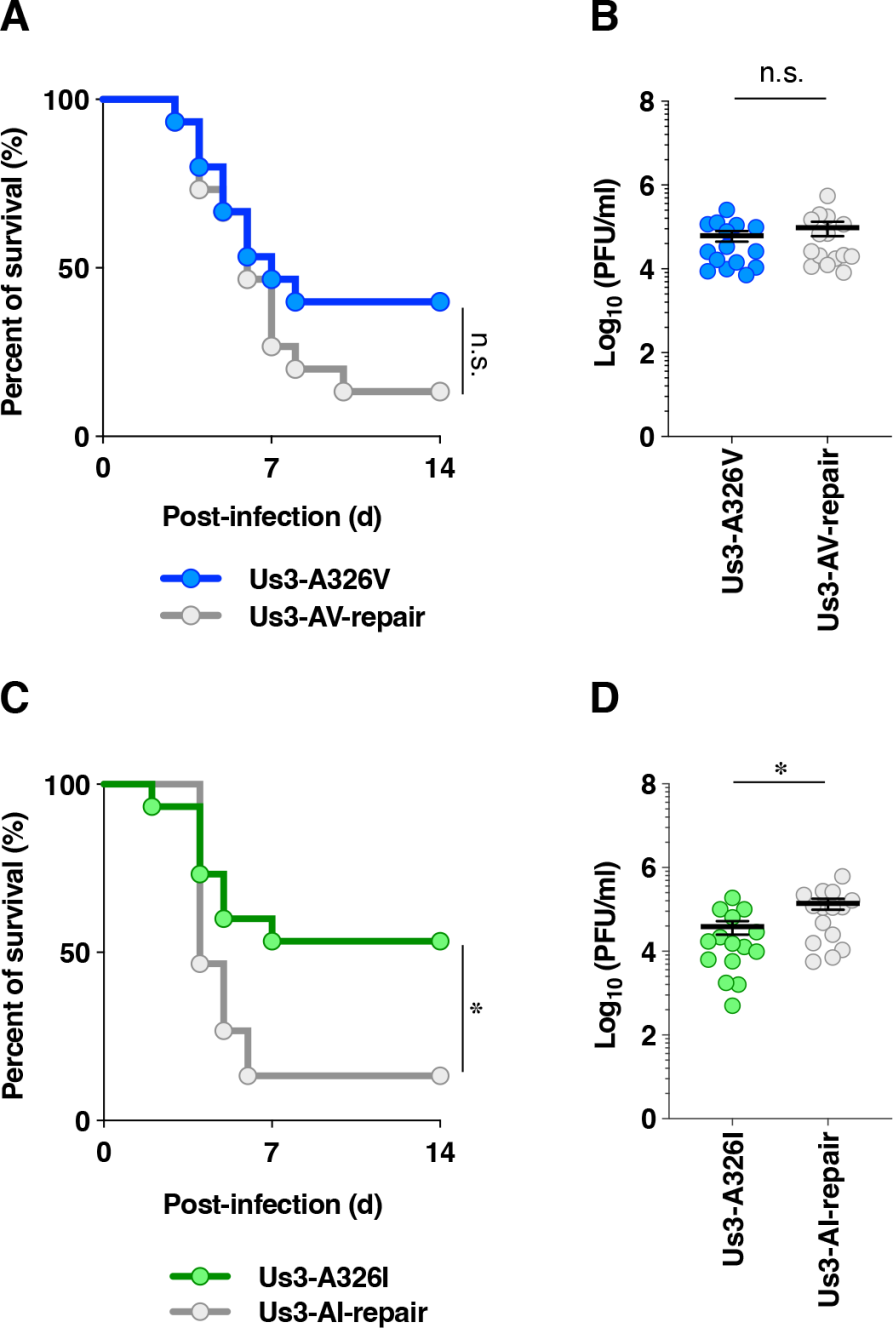
Effects of the A326V or A326I mutations in Us3 on the mortality rate and viral replication in the brains of mice following intracranial infection. (**A and C**) Three-week-old female mice were infected intracranially with 3 × 10^2^ PFU/head of YK801 (Us3-A326V) (*n* = 15) and YK802 (Us3-AV-repair) (*n* = 15) (**A**), or YK803 (Us3-A326I) (*n* = 19) and YK804 (Us3-AI-repair) (*n* = 18) (**C**), and were monitored for 14 days. Differences in the mortality rate of infected mice were analyzed statistically by the Log-rank test. ^*^; *P*  <  0.05, n.s., not significant. (**B and D**) Three-week-old female mice were infected intracranially with 3 × 10^2^ PFU/head of YK801 (Us3-A326V) and YK802 (Us3-AV-repair) (**B**), or YK803 (Us3-A326I) and YK804 (Us3-AI-repair) (**D**), and viral titers in the brains of infected mice 3 d post-infection were assayed. Each data point represents the virus titer in the brain of one mouse. The horizontal line and error bars indicate the mean ± SEM for each group. Each group consisted of 15 mice. Statistical significance was assessed by an unpaired two-tailed Student’s *t*-test. *; *P* < 0.05, n.s., not significant.

To examine the effects of the A326V and A326I mutations in Us3 on HSV-1 infection in the central nervous system (CNS) *in vivo*, 3-week-old female ICR mice were infected intracranially with 3 × 10^2^ plaque-forming units (PFU) of YK801 (Us3-A326V), YK802 (Us3-AV-repair), YK803 (Us3-A326I), or YK804 (Us3-AI-repair). The survival of infected mice was monitored for 14 days post-infection, and virus titers in infected mouse brains were quantified at 3 days post-infection. The survival rate of mice infected with YK801 (Us3-A326V) was slightly higher than that of mice infected with YK802 (Us3-AV-repair), although the difference did not reach statistical significance (Fig. 15A). In contrast, the survival rate of mice infected with YK803 (Us3-A326I) was significantly higher than that of those infected with YK804 (Us3-AI-repair) (Fig. 15C). In agreement with this result, titers in the brains of mice infected with YK801 (Us3-A326V) were comparable with those of mice infected with YK802 (Us3-A326V-repair) (Fig. 15B). In contrast, titers in the brains of mice infected with YK803 (Us3-A326I) were significantly lower than those in mice infected with YK804 (Us3-AI-repair) (Fig. 15D). These results indicated that a specific amino acid substitution at Us3 Ala-326 (A326I, but not A326V) significantly reduced viral replication and pathogenicity in the CNS of mice following intracranial infection.

**Fig 16:**
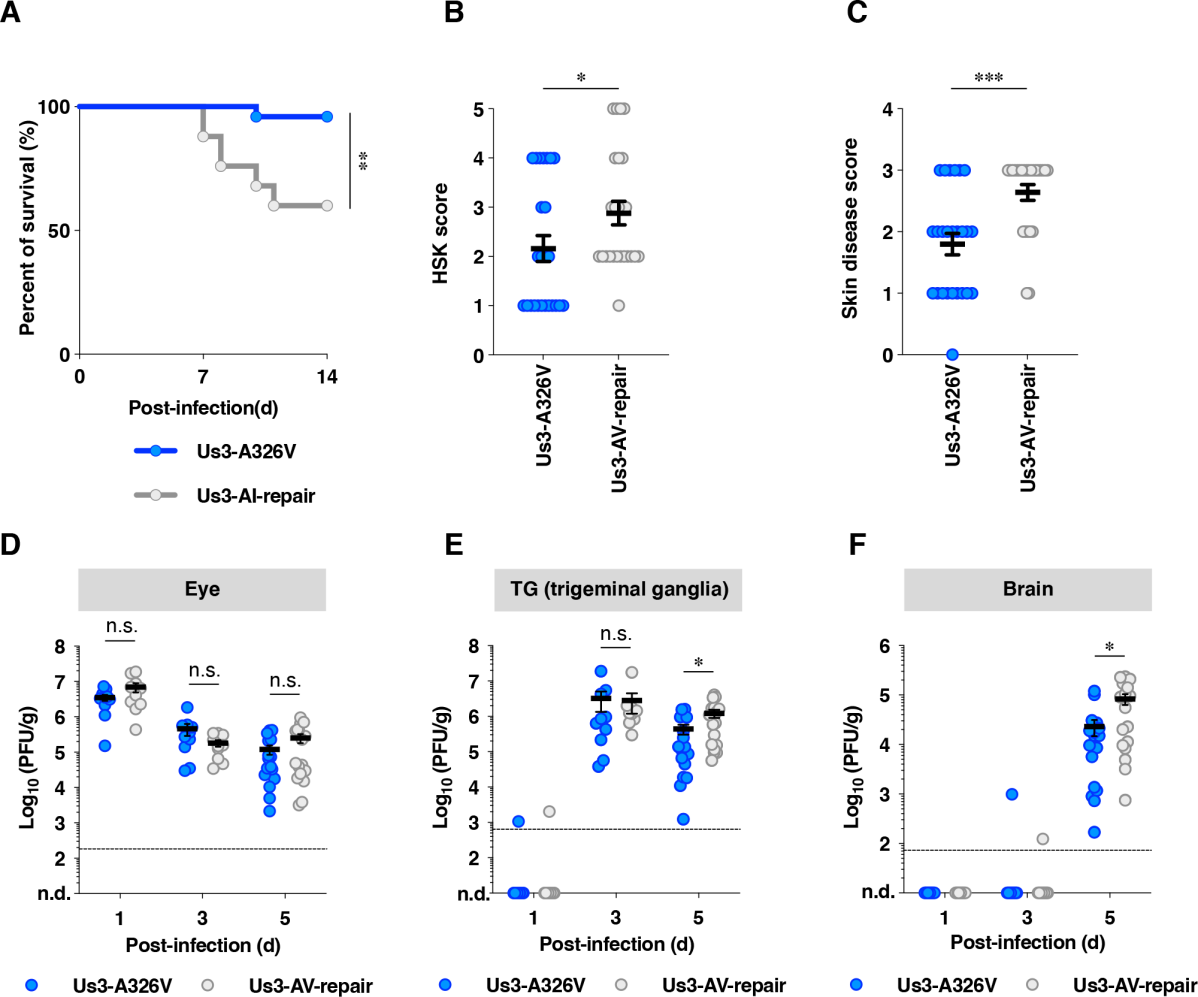
Effects of the A326V mutation in Us3 on pathogenic effects in and around the eyes, the mortality rate, and viral replication in mice following ocular infection. (A to C) Four-week-old female mice were infected ocularly with 1 × 10^5^ PFU/eye of YK801 (Us3-A326V) or YK802 (Us3-AV-repair). The survival rate of mice was monitored for 14 d post-infection (**A**). Statistical significance was assessed by Log-rank test. **; *P* < 0.01. At 6 days post-infection, the development of HSK (**B**) and periocular skin disease (**C**) was scored. Each data point represents the HSK score and periocular skin disease score of one mouse. The horizontal line and error bars indicate the mean ± SEM for each group. Each group consisted of 25 mice. Statistical significance was assessed by an unpaired two-tailed student’s *t*-test. ^*^; *P*  <  0.05, ^***^; *P*  <  0.001. (D to F) Four-week-old female mice were infected ocularly with 1 × 10^5^ PFU/eye of YK801 (Us3-A326V) or YK802 (Us3-AV-repair) (*n* = 15). Viral titers in the eyes (**D**), TGs (**E**), or brains (**F**) of infected mice at 1 and 3 days (*n*  =  10), and 5 days (*n*  =  17) post-infection were assayed. The detection limits of viral yields from eyes, TGs, and brains were 2.5 PFU/mL, 2.5 PFU/mL, and 25 PFU/mL, respectively. Because the mean weights of one eye, TG, and one brain were 0.038, 0.011, and 0.440 g, respectively, the detection limits were approximately 132.156, 478.160, and 56.805 PFU/g, respectively. The dashed lines indicate the limit of detection. n.d., not detected. Each data point represents the viral yield from one mouse. The horizontal line and error bars indicate the mean ± SEM for each group. Statistical significance was assessed by an unpaired two-tailed Student’s *t*-test. *; *P* < 0.05, n.s., not significant.

To clarify the *in vivo* significance of the A326V mutation in Us3, we employed a mouse ocular infection model. This model encompasses multiple stages of viral progression *in vivo*, including replication in epithelial tissues, infection within both the peripheral nervous systems (PNS) and CNS, and dissemination from epithelial tissues to the PNS and subsequently to the CNS (62). It also enables the evaluation of pathological manifestations in peripheral sites such as the eye and skin (29, 63). Compared with the intracranial infection model, the ocular model provides a more comprehensive view of viral phenotypes and is more sensitive to detecting subtle phenotypic differences associated with mutant viruses. Four-week-old female ICR mice were ocularly infected with 1 × 10^5^ PFU/eye of YK801 (Us3-A326V) or YK802 (Us3-AV-repair). The survival of infected mice was monitored for 14 days, and the development of herpes stromal keratitis (HSK) in their eyes and periocular skin disease at 6 days post-infection was evaluated. In addition, 4-week-old female ICR mice were infected ocularly with 1 × 10^5^ PFU/eye of YK801 (Us3-A326V) or YK802 (Us3-AV-repair). At 1, 3, and 5 days post-infection, mice were sacrificed, and virus titers in the eyes, trigeminal ganglia (TGs), and brains of the mice were measured. As shown in Fig. 16A through C, the survival rate of mice infected with YK801 (Us3-A326V) was significantly higher than that of mice infected with YK802 (Us3-AV-repair) (Fig. 16A). Furthermore, mice infected with YK801 (Us3-A326V) had significantly reduced severity of HSK and periocular skin disease compared with mice infected with YK802 (Us3-AV-repair) (Fig. 16B and C). Virus titers in the eyes of mice infected with YK801 (Us3-A326V) at 1, 3, and 5 days post-infection were similar to those of mice infected with YK802 (Us3-AV-repair) (Fig. 16D). Virus titers in the TGs of infected mice were barely detectable at 1 day post-infection, and both viruses reached comparable peak levels at 3 days post-infection (Fig. 16E). However, at 5 days post-infection, the viral titers of YK801 (Us3-A326V) were significantly lower than those of YK802 (Us3-AV-repair) (Fig. 16E). Similar to the reduction in viral titers in the TGs, viral titers in the brains of mice infected with YK801 (Us3-A326V) were also significantly lower at 5 days post-infection compared with YK802 (Us3-AV-repair) (Fig. 16F). These results indicated that the A326V mutation in Us3 significantly reduced viral replication in the TGs and subsequently in the brain. Consequently, the A326V mutation in Us3 markedly attenuated viral pathogenicity in mice following ocular infection. However, this mutation appeared to have minimal impact on viral replication in the eyes or viral entry into the TGs following ocular infection in mice.

## DISCUSSION

This study demonstrated that specific amino acid substitutions at Ala-326 in the A-loop of the HSV-1 Us3 PK significantly affected the phosphorylation levels of a specific subset of proteins mediated by Us3 in HSV-1-infected cells. These substrate-specific modulations of the Us3 mutants were in accord with previous observations in cellular serine/threonine PKs including LCK and dual-specificity tyrosine phosphorylation-regulated kinase 1A (DYRK1A), where alterations in the A-loop significantly influenced specific substrate interactions and substrate-specific phosphorylation events (52, 64). The mutations in Us3 Ala-326 reduced cell-to-cell viral spread in cell cultures and attenuated viral replication, pathogenicity, and/or pathogenic manifestations in mice. Thus, this study demonstrated a direct link between maintaining the proper fine-tuning of Us3-mediated phosphorylation across its target repertoire and the promotion of HSV-1 infection *in vitro* and *in vivo*. In agreement with this, it has been reported that HSV-1 lacking UL21, which antagonizes Us3-mediated phosphorylation via host phosphatases, upregulated the Us3-mediated phosphorylation of UL31, but not UL34, and reduced plaque size (61, 65). Conceivably, Us3 tightly and precisely regulates the phosphorylation levels of individual substrates within its repertoire during HSV-1 infection, and this overall regulation is likely critical for viral cell-to-cell spread *in vitro* and replication and pathogenicity *in vivo*. We noted that the level of phosphorylation at gB Thr-887 in lysates from cells infected with YK804 (Us3-A326I-repair) showed a downward trend compared with that from cells infected with wild-type HSV-1(F) (Fig. 3C and F). This observation raises the possibility that YK803 (Us3-A326I) may harbor secondary mutation(s) that could affect its phenotype(s) other than those examined in this study. Given that the presence of secondary mutation(s) cannot be completely excluded in any recombinant HSV-1 strain, direct comparisons between each mutant virus and its corresponding repair virus are essential for accurate interpretation of phenotypic differences. Taken together, our data provide supporting evidence for the conclusion that Us3 A326I mutation results in decreased phosphorylation of gB Thr-887, reduced viral cell-to-cell spread in cultured cells, and impaired viral replication and pathogenicity in an intracranial mouse infection model.

The intracranial infection of mice with HSV-1 can be used to study how viral replication can damage the CNS, whereas peripheral (e.g., ocular or vaginal) HSV-1 infection can be used to study multiple aspects of HSV-1 infection, including pathogenic manifestations at peripheral sites of infection, the capacity to invade the PNS including the TG from peripheral sites of infection, and the invasion of the CNS from the PNS. This study demonstrated that substitutions at Us3 Ala-326 with different amino acids affected the phosphorylation levels of most distinct Us3 target proteins and produced distinct phenotypes in mice following intracranial infection. The Us3 A326I mutation, unlike the Us3 A326V mutation, significantly impaired viral replication and pathogenicity in the CNS of mice following intracranial infection. This mutation altered the phosphorylation levels of p34, p39, and p49 in addition to those altered by the A326V mutation, suggesting that their phosphorylation is important in the regulation of viral CNS pathogenicity. We also showed that the Us3 A326V mutation, which significantly impaired pathogenic manifestations in and around the eyes, viral replication in the TGs and brains, and viral pathogenicity in mice following ocular infection, although the mutation had no effect on viral replication in eyes and viral entry into the TGs, altered the phosphorylation levels of p167, p144, and p37. Thus, phosphorylation of these three proteins might regulate multiple aspects of HSV-1 infection other than viral replication at the peripheral site and the capacity to invade the CNS from peripheral sites of infection. To date, the only Us3 substrates whose phosphorylation has been linked to HSV-1 infection *in vivo* are gB, Us3, VP13/14, and vdUTPase (24, 25, 29, 31). Among the six Us3 target proteins including p34, p37, p39, p49, p144, and p167 described above, p37 was suggested to be UL31, but the remaining five proteins may be new Us3 targets that are involved in viral replication and/or pathogenicity *in vivo*. Further studies to characterize the six Us3 targets described above will be of interest and provide further insights into the mechanisms by which Us3 regulates viral replication and pathogenicity *in vivo*. Moreover, it seems plausible that the A326V and A326I mutations in Us3 affect the phosphorylation of target proteins that are not detected by phospho-PKA substrate 100G7E and phospho-AKT substrate 110B7E antibodies, thereby contributing to the observed phenotypic differences.

Although the A-loop in cellular PKs is known to be critical for the regulation of PK activity, substrate specificity, and proper fine-tuning of phosphorylation across their substrate repertoire (6, 7), its role in viral PKs is unknown. To the best of our knowledge, this is the first study to report that the A-loop in viral PKs is important in fine-tuning the phosphorylation of a viral PK, HSV-1 Us3, as well as HSV-1 infection *in vitro* and *in vivo*. Ala-326 in the A-loop of Us3, targeted in our mutational analyses, is well conserved in the genera *Simplexvirus*, *Mardivirus*, and *Varicellovirus* in the subfamily *Alphavirinae* (Fig. 1E), suggesting its regulatory effects are conserved in Us3 homologs of the members in these genera. Of note, Gly-344 and Thr-345 in the A-loop of Us3 are conserved in members of the genera *Simplexvirus*, *Mardivirus*, *Varicellovirus,* and *Itovirus*, as well as in host cellular kinases PKA and AKT (Fig. 1A and E), which share similar substrate specificity with Us3 (16–19). Therefore, these residues might also be involved in regulating substrate specificity, rather than fine-tuning phosphorylation across the Us3 substrate repertoire, in these viral and cellular protein kinases. In contrast, the Ala residues at the DFG + 1 positions are much less conserved in host cellular PKs (1). A previous study reported that selective inhibitors of cellular PKs in the CLK subfamily can be developed by targeting the residue around the DFG motif (66). Therefore, targeting Ala at the DFG + 1 position in Us3 might be a novel strategy for developing selective inhibitors of Us3 and its homologs with minimal off-target effects on host cells.

## MATERIALS AND METHODS

### Phylogenetic analysis, sequence alignment, and logo generation

Amino acid sequences of Us3 homologs from the *Alphaherpesvirinae* subfamily registered in the ICTV databases (https://ictv.global/report/chapter/orthoherpesviridae/orthoherpesviridae/alphaherpesvirinae) were analyzed. Sequence alignments for each genus were conducted using the MUSCLE algorithm implemented in MEGA X software (67). The aligned sequences were then used to generate sequence logos using the WebLogo tool (https://weblogo.berkeley.edu) (68). These sequence logos illustrate the conserved residues within the A-loop region of Us3 homologs across different genera. The viruses and corresponding accession numbers for the sequences used in this analysis are provided in Table S1.

### Protein structure prediction and structural similarity analysis

Protein structures were predicted using AlphaFold3 (https://alphafoldserver.com/welcome) (54), and the resulting CIF files were converted to PDB format for subsequent analyses. The conversion was performed using PyMOL (version 2.5.7, https://pymol.org/installers/) with the following steps: (i) the CIF file was opened in PyMOL; (ii) “Export Molecule” was selected from the “File” menu; (iii) the target CIF file was chosen in the “Save Molecule” window, with the state set to “−1 (current)”; (iv) the “PDB” option was selected, enabling the “write CONECT records for all bonds” checkbox; and (v) the file was saved in PDB format. Structural similarity between two predicted protein structures was assessed using the TM-score (57). The PDB files of the protein structures were uploaded to the TM-align web server (https://zhanggroup.org/TM-align/), and TM scores were calculated to evaluate structural similarity according to the protocol provided by the tool.

### Cells and viruses

Simian kidney epithelial Vero cells and the HSV-1 wild-type strain HSV-1(F) were described previously (69–71). Human retinal pigment epithelial ARPE-19 cells were purchased from the American Type Culture Collection and maintained in DMEM/12 medium containing 10% fetal calf serum. The UL31-null HSV-1 YK720 (ΔUL31) and the repaired virus HSV-1 YK721 (ΔUL31-repair) were described previously (72). For experiments with YK720 (ΔUL31), wild-type HSV-1(F) and YK721 (ΔUL31-repair) viruses were propagated in UL31o-TetON-Vero cells described below.

### Construction of recombinant viruses

Recombinant viruses YK801 (Us3-A326V), encoding Us3 with valine substituted for Ala-326, YK803 (Us3-A326I), encoding Us3 with isoleucine substituted for Ala-326, and YK805 (Us3-K220M), encoding Us3 with methionine substituted for the lysine codon at position 220 (Lys-220) (Fig. 2) were generated by a two-step Red-mediated mutagenesis procedure using *Escherichia coli* GS1783 containing pYEbac102Cre as described previously (37, 62, 73–75), except the primers listed in Table 1 were used. Recombinant viruses YK802 (Us3-AV-repair), YK804 (Us3-AI-repair), and YK806 (Us3-KM-repair), in which the A326V, A326I, and K220M mutations in Us3 were repaired (Fig. 2), were generated by a two-step Red-mediated mutagenesis procedure as described previously (37, 75), except *E. coli* GS1783 containing the Us3-A326A, Us3-A326I, and Us3-K220M genomes were used with the primers listed in Table 1. The genotype of each recombinant virus was confirmed by PCR and sequencing.

**TABLE 1:** Oligonucleotide sequences for the construction of recombinant viruses

Recombinant virus	Oligonucleotide sequence (5′−3′)	Plasmid DNA template	*E*. *coli* GS1873 containing Hsv-1 BAC
Us3-A326V	5′-TATTAACACCCCCGAGGACATTTGCCTGGGGGACTTTGGTGTTGCGTGCT TCGTGCAGGGAGGATGACGACGATAAGTAGGG-3′	pEP-KanS (75)	*E*. *coli* GS1783/pYEbac102Cre (73, 74)
	5′-GGAAGGGGCTTGATCGGGAACCCTGCACGAAGCACGCAACACCAAAGTC CCCCAGGCAAACAACCAATTAACCAATTCTGATTAG-3′		
Us3-A326I	5′-TATTAACACCCCCGAGGACATTTGCCTGGGGGACTTTGGTATCGCGTGCT TCGTGCAGGGAGGATGACGACGATAAGTAGGG-3′	pEP-KanS (75)	*E*. *coli* GS1783/pYEbac102Cre (73, 74)
	5′-GGAAGGGGCTTGATCGGGAACCCTGCACGAAGCACGCGATACCAAAGTC CCCCAGGCAAACAACCAATTAACCAATTCTGATTAG-3′		
Us3-AV-repair / Us3-AI-repair	5′-TATTAACACCCCCGAGGACATTTGCCTGGGGGACTTTGGTGCCGCGTGCT TCGTGCAGGGAGGATGACGACGATAAGTAGGG-3′	pEP-KanS (75)	*E*. *coli* GS1783 containing the Us3-A326V/Us3-A326I genome (this study)
	5′-GGAAGGGGGCTTGATCGGGAACCCTGCACGAAGCACGCGGCACCAAAGT CCCCCAGGCAAACAACCAATTAACCAATTCTGATTAG-3′		
Us3-K220M	5′-TGACAGCAGCCACCCAGATTACCCCCAACGGGTAATCGTGATGGCGGGG TGGTACACGAGAGGATGACGACGATAAGTAGGG-3′	pEP-KanS (75)	*E*. *coli* GS1783/pYEbac102Cre (73, 74)
	5′-GCAGTCGCGCCTCGTGGCTCGTGCTCGTGTACCACCCCGCCATGATTACCC GTTGGGGGTCAACCAATTAACCAATTCTGATTAG-3′		
Us3-KM-repair	5′-TGACAGCAGCCACCCAGATTACCCCCAACGGGTAATCGTGAAGGCGGGG TGGTACACGAGAGGATGACGACGATAAGTAGGG-3′	pEP-KanS (75)	*E*. *coli* GS1783 containing the Us3-K220M genome (this study)
	5′-GTCGCGCCTCGTGGCTCGTGCTCGTGTACCACCCCGCCTTCACGATTACCC GTTGGGGGTCAACCAATTAACCAATTCTGATTAG-3′		

### Antibodies

Mouse monoclonal antibodies to gB (H1817) and β-actin (AC15) were purchased from Virusys and Sigma, respectively. Rabbit monoclonal antibodies to phospho-PKA substrate (100G7E) and phospho-AKT substrate (110B7E) were purchased from Cell Signaling Technology. Mouse monoclonal antibodies that specifically recognize gB with phosphorylated Thr-887 (gB-T887^P^) or Us3 with phosphorylated Ser-147 (Us3-S147^P^) were described previously (24, 29). Rabbit polyclonal antibodies to Us3 and UL31 were described previously (37, 76).

### Establishment of stable Vero cells with tetracycline-inducible UL31o expression

Vero cells were transduced with the supernatants of Plat-GP cells co-transfected with pMDG (48) and pRetroX-Tet3G (TaKaRa), selected with 1 mg/mL G418 (Wako), and further transduced with supernatants of Plat-GP cells cotransfected with pMDG and pRetroX-TRE3G-UL31o, which was synthesized (GenScript) (Table 2). For tetracycline-inducible (TetON) UL31o expression, after double selection with 5  µg/mL puromycin (Sigma) and 1 mg/mL G418, resistant cells were designated UL31o-TetON-Vero cells.

**TABLE 2:** Summary of synthesized plasmids

Constructed plasmid	Synthesized DNA sequence^*a*^
pRetroX-TRE3G-UL31o	ggatccgccaccatgtacgacacagatcctcacaggagaggctctaggccaggaccatatcacggcaagga gaggcgccggagccgcagctccgccgcaggaggcaccctgggcgtggtgagaagggcatccaggaagtc tctgccacctcacgccagaaagcaggagctgtgcctgcacgagagacagaggtacaggggactgttcgccg ccctggcacagacacccagcgaggagatcgccatcgtgaggtctctgagcgtgcctctggtgaagaccacac ccgtgtccctgcctttttgcctggaccagaccgtggccgataactgtctgacactgtctggcatgggctactatctg ggaatcggaggatgctgtcctgcatgcaatgcaggcgacggcaggttcgcagccaccagccgcgaggccct gatcctggcctttgtgcagcagatcaacacaatcttcgagcaccgggcctttctggcctccctggtggtgctggc cgacagacacaatgcccctctgcaggatctgctggcaggcatcctgggacagccagagctgttctttgtgcac accatcctgaggggaggaggagcatgcgacccaaggctgctgttctaccctgatccaacctatggcggccac atgctgtacgtgatctttccaggcacatctgcccacctgcactatcggctgatcgacagaatgctgaccgcatgtc caggatacaggttcgtggcacacgtgtggcagtccacatttgtgctggtggtgcgcaggaacgcagagaagcc aaccgacgcagagatcccaacagtgagcgccgccgatatctattgcaagatgcgggacatctccttcgatggc ggcctgatgctggagtaccagagactgtatgccaccttcgatgagtttccacccccttgagaattc

^*a*^ Restriction enzyme sites are underlined.

### Immunoblotting using the SimpleWestern platform

Protein expression was analyzed using the SimpleWestern platform (58, 59), Jess (ProteinSimple), which employs capillary electrophoresis for automated immunoassays, according to the manufacturer’s instructions. This platform provides highly linear quantification. Briefly, samples were prepared in SimpleWestern sample buffer, denatured at 95°C for 5 min, and loaded into the SimpleWestern capillary system. Subsequent procedures, including protein separation, antibody incubation, signal detection, and data processing, were performed automatically according to the standard protocol provided by the manufacturer. Protein quantification was conducted using the algorithm implemented in Compass software (ProteinSimple). Relative phospho-protein levels were normalized to the corresponding total protein or β-actin levels.

### Chemical treatments

The PKA inhibitor staurosporine (STS) was purchased from Selleck Chemicals (Texas, USA) and was used at a final concentration of 2 µM.

### Determination of plaque size

Plaque size was determined as described previously (74), except that a fluorescence microscope (BZ-X800, KEYENCE, Osaka, Japan) and the BZ-X800 Analyzer (KEYENCE) were used.

### Animal studies

For intracranial infection, female ICR mice (3 weeks) were purchased from Charles River and inoculated intracranially with 3 × 10² PFU of the indicated viruses as previously described (62, 77, 78). Mice were monitored daily, and mortality occurring between 1 and 14 days post-infection was attributed to the inoculated virus. Viral titers in mouse brains were determined by following established protocols (62, 77, 78). For ocular infection, 4-week-old female ICR mice were infected with 1 × 10⁵ PFU per eye of the indicated viruses as previously described (62, 74, 79, 80). The severity of HSK and periocular skin disease was scored using a previously established scale (62, 74, 79, 80). Mice were monitored daily, and mortality occurring between 1 and 14 days post-infection was attributed to the virus. At the indicated time points, infected mice were euthanized, and eyes, TGs, and/or brains were harvested. The wet weights of the tissues were measured, followed by sonication in 0.5 mL Medium 199 (Sigma) containing 1% fetal calf serum and antibiotics. The homogenized samples were then frozen at −80°C as previously described (62). Frozen samples were thawed and centrifuged, and viral titers in the supernatants were quantified using standard plaque assays on Vero cells (62, 80). These mouse models of HSV-1 infection are widely used, and the results, when using wild-type viruses, are highly reproducible (74). All animal experiments were performed in accordance with the Guidelines for the Proper Conduct of Animal Experiments, Science Council of Japan.

### Statistical analysis

Differences in the viral yields, plaque sizes, and relative intensities of phosphorylated proteins were analyzed statistically by ANOVA and Tukey’s test or an unpaired two-tailed Student *t*-test. Differences in the viral yields and disease scores of infected mice were analyzed statistically by an unpaired two-tailed Student *t*-test. Differences in the survival rates of infected mice were analyzed statistically by the Log-rank test. A *P*-value < 0.05 was considered statistically significant.

## Data Availability

The amino acid sequences used in this study are available from the National Center for Biotechnology Information. The viruses and their corresponding accession numbers for these genome sequences are listed in Table S1.

## SUPPLEMENTAL MATERIAL

Table S1 jvi.00400-25-s0001.pdf Accession numbers of Us3 homolog sequences used in this study.

ASM does not own the copyrights to Supplemental Material that may be linked to, or accessed through, an article. The authors have granted ASM a non-exclusive, world-wide license to publish the Supplemental Material files. Please contact the corresponding author directly for reuse.
