# Supplementary material for: Impact of the changes in substrate specificity of herpes simplex virus 1 protein kinase Us3 on viral infection in vitro and in vivo
Source: J Virol. 2025 Jun 30;99(7):e00400-25. doi: 10.1128/jvi.00400-25 (PMC12282182; doi:10.1128/jvi.00400-25)
Supplement: Table S1 — Accession numbers of Us3 homolog sequences used in this study. [file jvi.00400-25-s0001.pdf]

Table S1. Accession numbers of Us3 homologs sequences using in this study.

Virus	Accession Number	Link
Ateline alphaherpesvirus 1 (HVA1)	KY38563	https://www.ncbi.nlm.nih.gov/nuccore/KY385637
Bovine alphaherpesvirus 2 (BMV)	MT862163	https://www.ncbi.nlm.nih.gov/nuccore/MT862163
Cercopithecine alphaherpesvirus 2 (SA8)	AY714813	https://www.ncbi.nlm.nih.gov/nuccore/AY714813
Human alphaherpesvirus 1 (HSV1)	JN555585	https://www.ncbi.nlm.nih.gov/nuccore/JN555585
Human alphaherpesvirus 2 (HSV2)	JN561323	https://www.ncbi.nlm.nih.gov/nuccore/JN561323
Leporid alphaherpesvirus 4 (LHV4)	JQ596859	https://www.ncbi.nlm.nih.gov/nuccore/JQ596859
Macacine alphaherpesvirus 1 (BV)	AF533768	https://www.ncbi.nlm.nih.gov/nuccore/AF533768
Macacine alphaherpesvirus 2 (McAHV2)	KY628968	https://www.ncbi.nlm.nih.gov/nuccore/KY628968
Macacine alphaherpesvirus 3 (McAHV3)	KY628970	https://www.ncbi.nlm.nih.gov/nuccore/KY628970
Macropodid alphaherpesvirus 1 (MaAHV1)	KT594769	https://www.ncbi.nlm.nih.gov/nuccore/KT594769
Macropodid alphaherpesvirus 2 (MaAHV2)	MT900475	https://www.ncbi.nlm.nih.gov/nuccore/MT900475
Macropodid alphaherpesvirus 4 (MaAHV4)	MT900474	https://www.ncbi.nlm.nih.gov/nuccore/MT900474
Panine alphaherpesvirus 3 (ChHV)	JQ360576	https://www.ncbi.nlm.nih.gov/nuccore/JQ360576
Papiine alphaherpesvirus 2 (HPV2)	DQ149153	https://www.ncbi.nlm.nih.gov/nuccore/DQ149153
Teropodid alphaherpesvirus 1 (FBAHV1)	AB825953	https://www.ncbi.nlm.nih.gov/nuccore/AB825953
Pteropodid alphaherpesvirus 2 (PLAHV)	LC492974	https://www.ncbi.nlm.nih.gov/nuccore/LC492974
Saimiriine alphaherpesvirus 1 (HVS1)	HM625781	https://www.ncbi.nlm.nih.gov/nuccore/HM625781
Bovine alphaherpesvirus 1 (IBRV)	JX898220	https://www.ncbi.nlm.nih.gov/nuccore/JX898220
Bovine alphaherpesvirus 5 (BoAHV5)	AY261359	https://www.ncbi.nlm.nih.gov/nuccore/AY261359
Bubaline alphaherpesvirus 1 (BuAHV1)	KU936049	https://www.ncbi.nlm.nih.gov/nuccore/KU936049
Canid alphaherpesvirus 1 (CHV)	KT819633	https://www.ncbi.nlm.nih.gov/nuccore/KT819633
Caprine alphaherpesvirus 1 (CpAHV1)	MG989243	https://www.ncbi.nlm.nih.gov/nuccore/MG989243
Cercopithecine alphaherpesvirus 9 (SVV)	AF275348	https://www.ncbi.nlm.nih.gov/nuccore/AF275348
Cervid alphaherpesvirus 1 (CvAHV1)	MH036942	https://www.ncbi.nlm.nih.gov/nuccore/MH036942

Cervid alphaherpesvirus 2 (CvAHV2)	MH036943	https://www.ncbi.nlm.nih.gov/nuccore/MH036943
Cervid alphaherpesvirus 3 (CvAHV3)	MH036941	https://www.ncbi.nlm.nih.gov/nuccore/MH036941
Equid alphaherpesvirus 1 (EAV)	AY665713	https://www.ncbi.nlm.nih.gov/nuccore/AY665713
Equid alphaherpesvirus 3(EqAHV3)	KM051845	https://www.ncbi.nlm.nih.gov/nuccore/KM051845
Equid alphaherpesvirus 4 (EqAHV4)	AF030027	https://www.ncbi.nlm.nih.gov/nuccore/AF030027
Equid alphaherpesvirus 6 (AsHV1)	MT012704	https://www.ncbi.nlm.nih.gov/nuccore/MT012704
Equid alphaherpesvirus 8 (EqAHV8)	MF431611	https://www.ncbi.nlm.nih.gov/nuccore/MF431611
Equid alphaherpesvirus 9 (EqAHV9)	AP010838	https://www.ncbi.nlm.nih.gov/nuccore/AP010838
Felid alphaherpesvirus 1 (FVRV)	FJ478159	https://www.ncbi.nlm.nih.gov/nuccore/FJ478159
Human alphaherpesvirus 3 (VZV)	X04370	https://www.ncbi.nlm.nih.gov/nuccore/X04370
Monodontid alphaherpesvirus 1 (MoAHV1)	MF678601	https://www.ncbi.nlm.nih.gov/nuccore/MF678601
Phocid alphaherpesvirus 1 (PcAHV1)	MH509440	https://www.ncbi.nlm.nih.gov/nuccore/MH509440
Suid alphaherpesvirus 1 (PRV)	JF797218	https://www.ncbi.nlm.nih.gov/nuccore/JF797218
Anatid alphaherpesvirus 1 (DEV)	JF999965	https://www.ncbi.nlm.nih.gov/nuccore/JF999965
Columbid alphaherpesvirus 1 (PHV)	KX589235	https://www.ncbi.nlm.nih.gov/nuccore/KX589235
Gallid alphaherpesvirus 2 (MDV)	AF243438	https://www.ncbi.nlm.nih.gov/nuccore/AF243438
Gallid alphaherpesvirus 3 (GaAHV3)	HQ840738	https://www.ncbi.nlm.nih.gov/nuccore/HQ840738
Meleagrid alphaherpesvirus 1 (HVT)	AF291866	https://www.ncbi.nlm.nih.gov/nuccore/AF291866
Spheniscid alphaherpesvirus 1 (SpAHV1)	LT608135	https://www.ncbi.nlm.nih.gov/nuccore/LT608135
Cacatuid alphaherpesvirus 2 (CcAHV2)	MK360902	https://www.ncbi.nlm.nih.gov/nuccore/MK360902
Gallid alphaherpesvirus 1 (ILTV)	JN596962	https://www.ncbi.nlm.nih.gov/nuccore/JN596962
Psittacid alphaherpesvirus 1 (PDV)	AY372243	https://www.ncbi.nlm.nih.gov/nuccore/AY372243
Psittacid alphaherpesvirus 5 (PsAHV5)	MK955929	https://www.ncbi.nlm.nih.gov/nuccore/MK955929
Chelonid alphaherpesvirus 5 (FPTHV)	HQ878327	https://www.ncbi.nlm.nih.gov/nuccore/HQ878327
Testudinid alphaherpesvirus 3 (TeHV3)	KM924292	https://www.ncbi.nlm.nih.gov/nuccore/KM924292
